# New and Rare Taxa of *Lepidoziaceae* (*Marchantiophyta*) in East Indochina (Southeast Asia)

**DOI:** 10.3390/plants15071136

**Published:** 2026-04-07

**Authors:** Vadim A. Bakalin, Ksenia G. Klimova, Elena V. Kushnevskaya, Van Sinh Nguyen, Hung Manh Nguyen, Alen K. Eskov, Nikolay G. Prilepsky, Anna S. Kartasheva, Seung Se Choi

**Affiliations:** 1 Laboratory of Cryptogamic Biota, Botanical Garden-Institute Far Eastern Branch of the Russian Academy of Sciences, Makovskogo Street 142, Vladivostok 690024, Russia; ksenia.g.klimova@mail.ru; 2 Department of Geobotany and Plant Ecology, Saint Petersburg State University, Universitetskaya Embankment 7/9, Saint Petersburg 199034, Russia; elly.kushn@gmail.com; 3 Department of General Ecology, Anatomy and Plant Physiology, Saint Petersburg State Forest Technical University, Institutskaya Street 5, Saint Petersburg 194021, Russia; 4 Institute of Biology, Graduate University of Science and Technology, Vietnam Academy of Science and Technology, Ha Noi 10072, Vietnam; nh.manhiebr@gmail.com; 5 Department of Plant Ecology and Geography, Faculty of Biology, Moscow State University, Leninskiye Gory 1–12, Moscow 119234, Russia; a.k.eskov@yandex.ru (A.K.E.); nprilepsky@mail.ru (N.G.P.); 6 Botany Department, State Museum of Natural History, Rosenstein 1, 70191 Stuttgart, Germany; kartasheva-2000@mail.ru; 7 Team of National Ecosystem Survey, National Institute of Ecology, Seocheon 33657, Republic of Korea

**Keywords:** *Arachniopsis*, *Bazzania*, *Lepidozia*, *Neolepidozia*, *Tricholepidozia*, distribution, Vietnam, Laos, Cambodia, liverworts, *Hepaticae*, liverwort taxonomy, bryophyte diversity, floristic survey

## Abstract

Ongoing studies on the Lepidoziaceae in East Indochina have yielded new information on the distribution and morphology of a number of family representatives. This study aimed to provide new data in this regard. The latter task looks quite justified, taking into account the fact that East Indochina houses a notable portion of the worldwide Lepidoziaceae diversity, especially in the genus *Bazzania*. The materials for the paper were 48 specimens collected throughout East Indochina. The cited specimens contain 18 taxa, discussed in respect of their ecology, distribution, and morphology. All the taxa discussed in this paper are supplemented with illustrations, and their morphological descriptions based on the Indochinese materials are also included in most cases. One taxon (*Bazzania appendiculata* subsp. *cambodiana* subsp. nov.) is described as new-for-science. Six species are new to Indochina, three species are new to East Indochina, one species is new to Vietnam, and three species are new to Cambodia. A comparison of the currently known taxonomic diversity with that of Malaysia, which borders East Indochina, reveals that the diversity of Lepidoziaceae in East Indochina is still clearly understudied, and further research is likely to yield new discoveries. The final target in this field is the creation of a thorough taxonomic revision of the family in this region in the future.

## 1. Introduction

Lepidoziaceae Limpr. is a fairly large liverwort family, with 754 taxa accepted in [1]. Many of the taxa listed in [1] are regarded as doubtful or having knowledge problems. Several taxa have been further (after [1] was published) described or reevaluated [2,3,4,5,6,7]. Many Lepidoziaceae representatives are large plants and have attracted attention for a long time. The most taxonomically diverse within the family is the genus *Bazzania* Gray. The first revision of the latter was published over 180 years ago, when it was segregated under the name *Mastigobryum* (Nees) Lindenb. et Gottsche [8]. Already cited authors noted the complex nature of this genus and discussed 54 species. The greatest diversity was noted on Java Island and ‘India orientali continenti’ (l.c. 214–215). The first worldwide monograph of the genus (under the name *Mastigobryum*) was also soon published thereafter [9].

The main diversity within this family in the Southeast Asian mainland (≈Indochina Peninsula) pertains to the genera *Bazzania* and *Lepidozia* (Dumort.) Dumort. A similar situation occurs in Malesia, adjacent to Indochina to the east. In Indochina plus Malesia and Melanesia, widely interpreted as Southeast Asia (https://en.wikipedia.org/wiki/Southeast_Asia, ≈Indochinese, Malaysian and Papuasian Floristic Regions by Takhtajan [10] (accessed on 15 January 2026)), a significant number of studies have dealt with these genera.

Among the earliest key works dealing with Lepidoziaceae, those of Sande Lacoste [11,12] are particularly noteworthy. They provided detailed illustrations that were unusual for their time. In the second half of the 20th century, as new data accumulated, a significant shift in the knowledge of Southeast Asian Lepidoziaceae occurred due to Mizutani [13,14,15]’s and Kitagawa [16,17,18,19]’s efforts. An important event in the knowledge of the taxonomy of the family occurred at the beginning of the first decade of the 21st century, because of Cooper et al. [20,21,22]. They published a number of papers on the molecular phylogeny of the family and provided corresponding nomenclatural changes, including the segregation of new genera. Almost simultaneously, a number of works on the diversity of the family began (and continue) to appear both in the region under consideration and in adjacent countries: China, Malaysia, Indonesia, and the Philippines [7,23,24,25,26,27]. Updating earlier studies on *Bazzania* in Vietnam [28], Pócs contributed significantly to knowledge on the genus in the country [6,29,30]. Our team has published studies on type specimens [31] and a checklist of *Bazzania* for Pacific Asia, with a special focus on Vietnam [3].

Despite the listed efforts, *Lepidoziaceae* are still far from being well understood in East Indochina. The previously obtained data on the diversity distribution patterns of *Bazzania* in Vietnam [3] may apparently be extrapolated to all Lepidoziaceae. Overall, the situation is rather uncertain: most of the data pertain to the northern part of the country; in the central part of the country, most of the data are from one to two provinces only; in the south of the country, very little information is available. In other countries of East Indochina, such as Laos and Cambodia, the situation is even worse. The information available for Cambodia may not be adequate, but is nonetheless noteworthy: *Acromastigum* A. Evans—2 species, *Bazzania*—10, *Lepidozia*—1, *Neolepidozia* Fulford et J. Taylor—1, *Tricholepidozia* (R.M. Schust.) E.D. Cooper—1 [32,33]. The current data for Laos, where only five species of *Bazzania* and one *Kurzia* species are known, likely do not reflect the true biodiversity [34,35]. At least the northern flank of Laos should be quite rich in this regard, owing to its high landscape diversity, including mountains reaching almost 3000 m a.s.l. and its proximity to the Sino-Himalayas—a unique location for Lepidoziaceae as well [13]. A discussion on this issue is provided by Bakalin et al. [35]. Therefore, the data on Lepidoziaceae distribution in East Indochina await elaboration.

Furthermore, we encountered a situation where, for a number of seemingly relatively widespread species, the available literature contains few, outdated, or no morphology illustrations or descriptions. Moreover, the data concerning species ecology, including habitat and associated plant communities, are almost absent for most of the taxa. This inspired us to conduct the present study, which includes a discussion (in most cases accompanied with morphological descriptions) and illustrations of taxa in the family that are (1) new to the study region, (2) rare in East Indochina, or (3) likely not rare but lack modern descriptions and illustrations in the available literature based on Southeast Asian specimens; therefore, these species are likely overlooked because of possible erroneous identifications.

The present account is the result of the collective effort of a number of researchers from various institutions in Vietnam, Russia and the Republic of Korea who have worked in East Indochina, including Laos, Cambodia, and Vietnam. This work was carried out to increase the availability of data on the family Lepidoziaceae and should contribute to the further compilation of a comprehensive revision of the family in East Indochina.

## 2. Results

In total, 48 specimens of 18 taxa were studied. Taxonomically, the studied specimens belong to the following genera: *Arachniopsis* Spruce (1 species), *Bazzania* (11 species and 1 subspecies), *Lepidozia* (2 species), *Neolepidozia* (2 species), and *Tricholepidozia* (1 species).

A summary of the treated Lepidoziaceae taxa is presented in Table 1, except for two taxa of *Neolepidozia*, for which status is unclear.

This study revealed one taxon new-for-science (*Bazzania appendiculata* subsp. *cambodiana*), six species new to Indochina (*Arachniopsis major* Herzog, *Bazzania commutata* (Lindenb. et Gottsche) Schiffn., *B. fleischeri* (Steph.) Abeyw., *Lepidozia holorhiza* (Reinw., Blume et Nees) Nees, *L. miqueliana* Sande Lac., *Tricholepidozia semperiana* (Steph.) E.D. Cooper), two species new to East Indochina (*B. callida* (Sande Lac. ex Steph.) Abeyw., *B. paradoxa* (Sande Lac.) Steph.), twospecies new to Vietnam (*B. albifolia* Horik., *B. appendiculata* (Mitt.) S. Hatt.), three species new to Cambodia (*Bazzania erosa* (Reinw., Blume et Nees) Trevis., *B. intermedia* (Gottsche et Lindenb.) Trevis., *B. vittata* (Gottsche) Trevis), the distribution was updated, and taxonomic notes were provided for four species (*Bazzania adnexa* (Lehm. et Lindenb.) Trevis., *B. recurva* (Mont.) Trevis., *Neolepidozia papulosa* (Steph.) Fulford et J. Taylor and *N. wallichiana* (Gottsche) Fulford et J. Taylor). Below, we provide the taxonomic treatment of all taxa treated in this study.

### Taxonomic Treatment

*Arachniopsis major* Herzog, Trans. Brit. Bryol. Soc. 1 (4): 294, 1950

*Telaranea major* (Herzog) J.J.Engel et G.L.Merr., Fieldiana, Bot. (n.ser.) 44: 165, 2004

Descriptions: [36].

Illustrations: [37] (Figures 12 and 13), [36,38] (Figure la,b).

**Figure 1:**
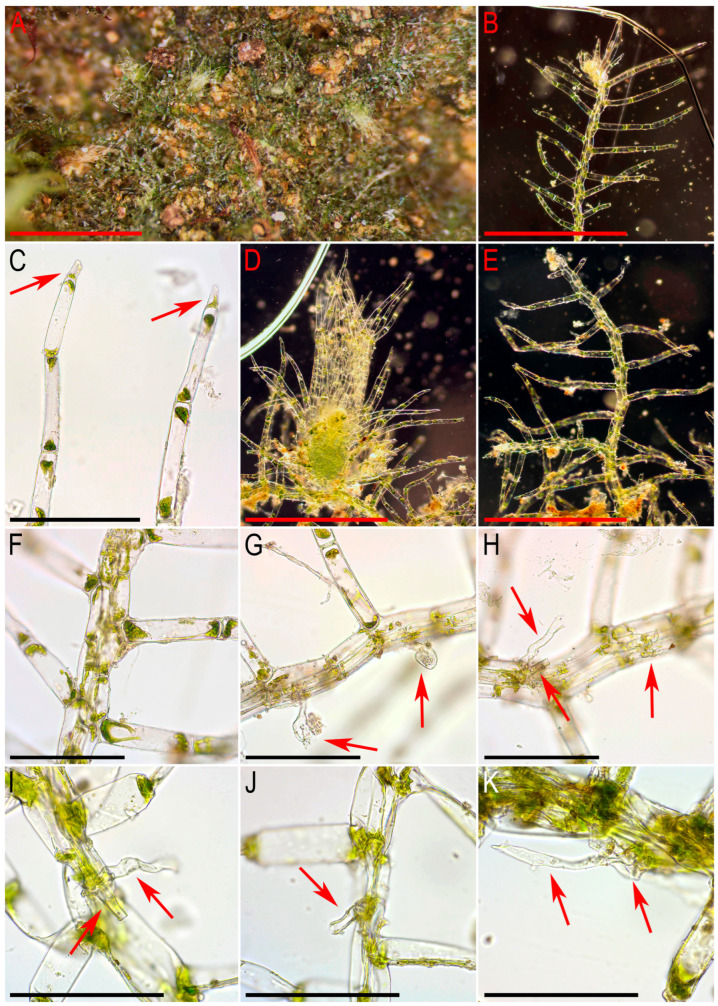
*Arachniopsis major* Herzog.: (**A**) part of mat in dry condition; (**B**,**E**) shoots with uniseriate cilia-like leaves; (**C**) upper parts of leaves, with terminal triangular cells (indicated by red arrows); (**D**) mature perianth with young sporophyte inside; (**F**) stem with leaf bases, dorsal view; (**G**–**K**) short paired rhizoids, except (**J**) where not paired (indicated by red arrows). (**B**,**D**,**E**) photographed with dark field option. Scales: 2 mm for (**A**); 500 µm for (**B**,**D**,**E**); 100 µm for (**C**,**F**–**K**). All from Fok23#8 kamen kushn8 (VBGI).

Description. Plants very delicate, closely attached to the substrate, easily fragmenting when detaching from the substrate, pale greenish, 3–5 mm long and 0.5–0.9 mm wide sparsely branched (branches *Frullania*-type, instead complete the leaf, Figure 1E), also merely commonly ventrally branched, with ventral branches soon becoming ‘normal’ in size. Stem cross section of 4 cells only: dorsal cells in two rows, 45–75 × (17–)20–25 µm, thin-walled, its vestigial trigones, cuticle smooth; ventral segments with two rows of cells, 30–100 × 10–15 µm, thin-walled, with vestigial trigones, cuticle smooth. Rhizoids as the underleaf continuation. Leaves reduced to one uniseriate cilia 4–5 cells long, with cells ca. 80–120 µm long and (20–)25–27(–30) µm wide, rectangular, somewhat narrower to the apex where only (12–)15–17 µm wide, except of the terminal cell that is always triangular, 12–15 × 7–10 µm; cells thin-walled throughout, trigones vestigial to very small, concave, cuticle virtually smooth, leaves situated between every second cell of the dorsal stem segment. Underleaves were not observed, where they should be we found only single (may be the second was simply broken there) or paired rhizoids instead, underleaves if they were present seem easily deciduous. Perianth on short ventral branch, suddenly turned vertically, with two rings of bracts and bracteoles; the outer ring has bracts with paired segments without distinct discus and with reduced bracteoles, but densely rhizogenous in that place; the inner ring has bracts with four lobes, each lobe two cells wide in the base, leaf lobes adjacent each to another, but discus is unclear. Perianth tubular, with undivided part 300–400 µm long and ca. 250 µm wide, continuing above as not branched cilia 250–350 µm long, cilia in perianth mouth similar to leaf segments or slightly narrower; only one archegonium per perianth was seen (the only one mature perianth was studied and the data may not be reliable).

Illustrations in the present paper: Figure 1.

Comment. This is the only species of *Arachniopsis* occurring in the nearest locations to the Indochina Peninsula (although over 1500 km away), while other similar taxa such as *Arachniopsis diacantha* (Mont.) M. Howe and *Arachniopsis coactilis* Spruce occur in tropical America and southern Africa, respectively [39]. Among the regional representatives of *Lepidoziaceae*, the species is easily distinguished by its leaves, reduced to single-row cilia, and a stem consisting of only four cells in cross-section. The plants reach 0.9 mm in width, thus exceeding most representatives of *Kurzia* and *Tricholepidozia* from *Lepidoziaceae*, as well as from other families with linearly lobed leaves, such as *Blepharostoma* (L.) Dumort. According to available data, this is the first known occurrence of this species in Indochina and in the Asian mainland. However, given the general patterns of distribution of the species, this discovery should be expected.

Our treatment of *Arachniopsis* as a genus separate from *Telaranea* Spruce ex Schiffn. contradicts both Engel and Merrill [39] and the subsequent world liverwort checklist by Söderström et al. [1]. However, as pointed out by Cooper et al. [20], the nomenclatural type of *Archniopsis* has not been sequenced, as with the nomenclatural type of *Telaranea*, whereas the latter genus shows clear paraphyly, contradicting the treatment by Engel and Merrill [39] based entirely on morphology. The species similar to the nomenclatural type of *Telaranea*, *T. herzogii* (E.A. Hodgs.) E.A. Hodgs., was placed in the clade *Zoopsis* III, while *Telaranea major* (=*Arachniopsis major*) is placed either in the clade ‘*Zoopsis* I’ or in the clade ‘*Zoopsis* II’. Moreover, the phylogenetic relationship of *Arachniopsis major* to *Telaranea coactilis* (Spruce) J.J. Engel et G.L. Merr. (*Arachniopsis* type species) is unknown. In addition, *Arachniopsis major* was actually found to be paraphyletic [20]. As Cooper et al. [20] (p. 500) note, “Nevertheless, it is clear from the current phylogeny that *Telaranea* species with reduced stem and leaf anatomy exhibit a convergent morphology, and more complete taxonomic sampling will be necessary before taxonomic changes are made.” A year later, Cooper et al. [21] confirmed the previous results, which placed ‘*Telaranea major’* in the clade ‘*Zoopsis* I’. Thus, there is no certainty as to which genus *Arachniopsis major* actually belongs (*Telaranea* s.str., *Zoopsis* Hook.f. ex Gottsche derivative, *Arachniopsis* s. str., or its own genus not described yet). In light of these data, we continue to maintain the generic status of *Arachniopsis*.

Distribution. The distribution of the species stretches from South Asia (Sri Lanka), East Indochina (new report), through Malesia and Melanesia to Oceania (eastward to Vanuatu) [39,40].

Ecology. We were not able to find data on habitat in the available sources. Our specimen was collected from a small stone in an evergreen tropical mountain forest with *Fokienia hodginsii* (Dunn) A. Henry et H.H. Thomas and *Pinus krempfii* Lecomte slightly above 1500 m a.s.l.

Specimen examined: VIETNAM. Central Highlands, Lâm Đồng Province, Lạc Dương District (12.181147° N 108.689575° E), 1532 m a.s.l., evergreen tropical mountain forest with *Fokienia hodginsii* (Dunn) A.Henry et H.H.Thomas and *Pinus krempfii* Lecomte, on small stone, may be sandstone or siltstone, 28 October 2023, E.V. Kushnevskaya, Fok23#8 kamen kushn8 (VBGI).

*Bazzania adnexa* (Lehm. et Lindenb.) Trevis., Mem. Reale Ist. Lombardo Sci. (Ser. 3), C. Sci. Mat. 4 (13): 414, 1877

Descriptions: [27,41,42].

**Figure 2:**
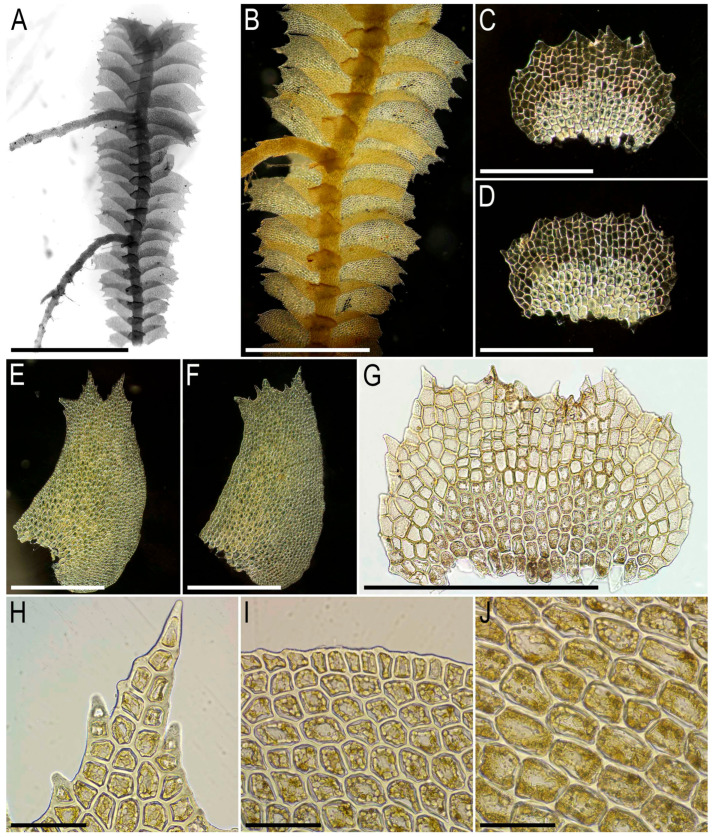
*Bazzania adnexa* (Lehm. et Lindenb.) Trevis.: (**A**,**B**) shoots with ventral scale-like leaved ‘flagella’, ventral view; (**C**,**D**,**G**) underleaves; (**E**,**F**) leaves; (**H**) leaf lobe cells; (**I**) cells of leaf margin; (**J**) midleaf cells. (**B**–**D**,**E**,**F**) are photographed with dark field option. Scales: 2 mm for (**A**); 1 mm for (**B**); 500 µm for (**E**,**F**); 300 µm for (**C**,**D**,**G**); 50 µm for (**H**–**J**). All from DAK6 Valezh1 kushn1 (VBGI).

Illustrations: [26] (Figure 4), [27] (Figure 2), [41,42] (Figure 4).

Description. Plants pale greenish to yellowish greenish, merely soft, in loose patches, sparsely branched, ventral scale-like leaved ‘flagella’ common, 6–20 mm long and 1.1–2.0 mm wide. Rhizoids not seen in normally leaved shoots, relatively abundant in terminal parts of ventral scale-like leaved ‘flagella’, where from the stem in the areas adjacent just below to the modified leaves and underleaves, colorless to brownish, mostly united into loose, obliquely spreading fascicles. Leaves obliquely inserted, dorsally insertion line arcuate, in moist conditions obliquely oriented and suberect spreading, without the teeth on the both ventral and dorsal bases, imbricate, covering to ½ of above situated leaf, obliquely ovate, sharply 3–lobed with several or solitary accessory teeth those are most evident in the lobed zone, when flattened in the slide 700–1100 × 500–600 µm. Midleaf cells considerably larger than at the margins, but not forming ‘vitta-like’ zone, 25–45 × 20–30 µm, thin-walled, trigones mostly moderate in size, concave to slightly convex, cuticle smooth; dorsal margin cells 7–13 µm, thick-walled, with small concave trigones, cuticle smooth to indistinctly punctate. Underleaves distant, plane to loosely concave, obliquely spreading, connate with the leaves in the both sides, when flattened in the slide transversely ellipsoidal, 200–300 × 350–500 µm, with margin composed by hyaline thin-walled and trigone-less cells, while from the middle part and below cells are chlorophyllose, more stout and bearing trigones, cuticle verrucose in hyaline part and virtually smooth below.

**Figure 3:**
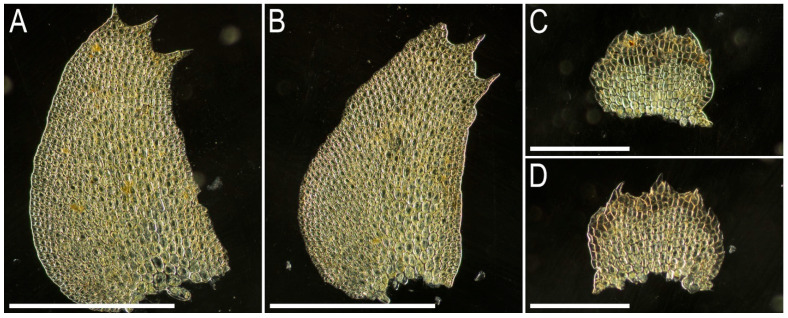
*Bazzania adnexa* (Lehm. et Lindenb.) Trevis.: (**A**,**B**) leaves; (**C**,**D**) underleaves. All are photographed with dark field option. Scales: 500 µm for (**A**,**B**); 300 µm for (**C**,**D**). (**A**–**D**) from DAK6 kamen3 kushn2 (VBGI).

Illustrations in the present paper: Figure 2 and Figure 3.

Comment. Zhou et al. [26] compare *Bazzania adnexa* with *B. japonica* (Sande Lac.) Lindb. These two species are indeed quite similar in the underleaf and leaf connation, but may be easily differentiated by the following traits: (1) presence of hyaline underleaf margin (versus its complete absence in *B. japonica*); (2) leaves are wider and only obliquely ovate in *B. adnexa*, compared with the oblong leaf shape with subparallel sides in its upper part in *B. japonica* and evident from the type-derived photographs and figures published in [2].

The species is somewhat morphologically similar to *B. intermedia*, from which it differs in terms of the smaller size of the plants and the underleaves being wider than they are long (versus longer than wide in *B. intermedia*). One more morphologically related taxon is *B. fleischeri* (discussed below) which differs from *B. adnexa* in its larger plants with distinctly developed darker (brownish) colored zone along the leaf margin composed by small cells with thick walls. Moreover, *B. fleischeri* has underleaves with the chlorophyllose zone persistent only very near the base, compared with *B. adnexa* where the chlorophyllose zone extends at least to the underleaf middle.

Pócs [29] reported this species for Vietnam, as presented in his Figure 1A,B. These figures provide photographs of one underleaf and the upper part of one leaf. Both do not correspond well with the description and illustration published before in [27,41,42]. The picture of the underleaf in [29] seems longer than it is wide and the leaf looks as if it’s going from the wide base, which is at least different from the plants we see in the literature and our collections. Therefore, we cannot regard the report as undoubtedly true regarding *B. adnexa*.

*Bazzania adnexa* is a mostly Australasian species and the identity of Chinese and Indochinese plants with Australasian providence was never tested, remaining a large field for speculations including: (1) whether the Indochinese and Australian plants are genetically identical, (2) whether *B. adnexa*-named plants collected in Indochina and China are genetically different from *B. intermedia* or simply the ecological deviation of the latter. It is worth mentioning that the Vietnamese location is situated at approximately the same latitude as the Chinese one (Hainan, cf. Zhou et al. [26,27]), and the plants from these two sites are most likely conspecific.

Distribution. East Asia (China: Hainan), Indochina (Vietnam), Melanesia (New Caledonia) Australasia (Australia, New Zealand) [26,27,41,42].

Ecology. In China, the species is known from decaying wood, trunks and branches in evergreen forests near 1100 m alt. (Hainan) [26]. Far away, ca. 8000 km to the southeast in New Caledonia, the species is occurring on decaying wood and rocks at elevations ca. 400–1000 m a.s.l. [42] thus in essentially the same habitat. The habitats in Vietnam cover decaying wood, tree trunk bases and small stones in tropical evergreen forests slightly above 1000 m a.s.l.

Specimen examined: VIETNAM. Central Highlands, Gia Lai Province, Kbang District (14.490503° N 108.56661° E), 1030 m a.s.l., evergreen tropical forest with substantial share *Dacrydium elatum* (Roxb.) Wall. ex Hook, on wood of fallen suspending trunk, 12 March 2022, E.V. Kushnevskaya, DAK6 Valezh1 kushn1 (VBGI).

The species is quite polymorphous especially in the leaf apical part accessory tooth density and hyaline zone of underleaf size. The following deviations may be recognized:

Specimen DAK6 kamen3 kushn2—leaves almost edentate, while underleaves are characteristic.

Specimen examined for deviations: VIETNAM. Central Highlands, Gia Lai Province, Kbang District (14.490503° N 108.56661° E), 1030 m a.s.l., evergreen tropical forest with substantial share *Dacrydium elatum*, on small stone, may be sandstone or siltstone, 12 March 2022, E.V. Kushnevskaya, DAK6 kamen3 kushn2 (VBGI).

*Bazzania albifolia* Horik., J. Sci. Hiroshima Univ., Ser. B, Div. 2, Bot. 2: 195, 1934

= *Bazzania semiopaca* N. Kitag. J. Hattori Bot. Lab. 30: 261. f. 5 1967

Descriptions: [27,43], ref. [16] (under *B. semiopaca* N. Kitag.).

Illustrations: [43] (Plate 16, Figures 22–30), [16,27] (Figure 5).

**Figure 4:**
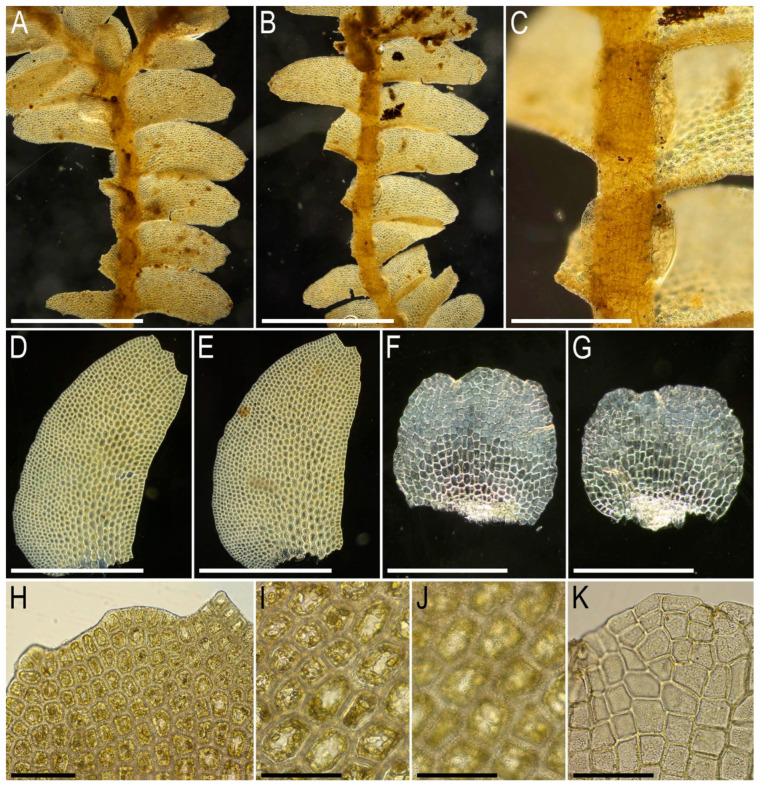
*Bazzania albifolia* Horik.: (**A**) shoot, dorsal view; (**B**) shoot, ventral view; (**C**) underleaves on a part of a shoot; (**D**,**E**) leaves; (**F**,**G**) underleaves; (**H**) leaf apex cells; (**I**,**J**) midleaf cells; (**J**) verruculose leaf cuticle in midleaf area; (**K**) verruculose leaf cuticle of underleaf apex. (**A**–**G**) are photographed with dark field option. Scales: 1 mm for (**A**,**B**); 500 µm for (**D**,**E**); 300 µm for (**C**,**F**,**G**); 50 µm for (**H**–**K**). (**A**–**C**) from isotype of *B. semiopaca* NICH-279771 (NICH), (**D**–**K**) from DAK6 Melky debris kushn4 (VBGI).

**Figure 5:**
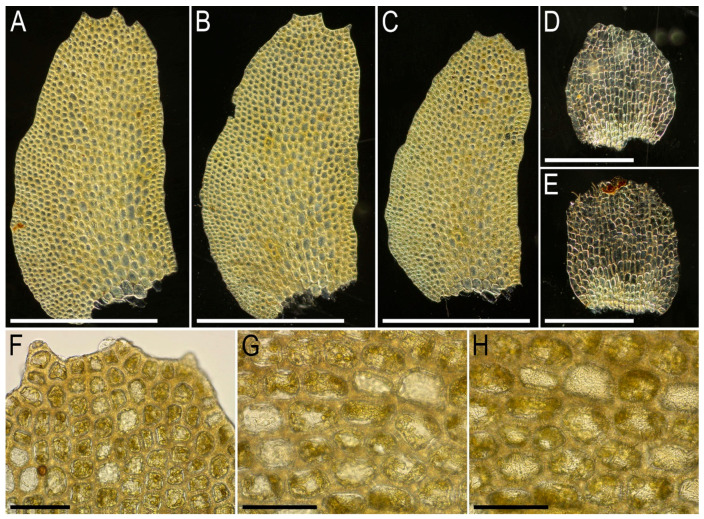
*Bazzania albifolia* Horik.: (**A**–**C**) leaves; (**D**,**E**) underleaves; (**F**) leaf apex cells; (**G**) midleaf cells; (**H**) verruculose leaf cuticle of midleaf area. (**A**–**E**) are photographed with dark field option. Scales: 500 µm for (**A**–**C**); 300 µm for (**D**,**E**); 50 µm for (**F**–**H**). All from Cam-81-33-11 (VBGI).

Illustrations in the present paper: Figure 4 and Figure 5.

Comment. The species was comprehensively described in the sources cited above. As it was circumscribed by Kitagawa [16] under the name *B. semiopaca*, and is morphologically most similar to the species *B. asperrima* Steph. The former is, however, different in its smaller size and only shortly divided leaves. The vast majority of our studied materials correspond very well to the original descriptions of *B. albifolia* and *B. semiopaca,* as well as the type of the latter (isotype in NICH-279771!). The basic features of *B. albifolia* are the hyaline underleaves, densely verruculose leaf cuticle and only shortly lobed leaves. There are, however, some deviations. One of them is provided by Cam-81–33–11, whose plants are characterized by more sharply lobed leaves, although the leaf ‘lobation’ is in any case much shorter than it should be in the ‘true’ *B. asperrima*. Whether the sympatric distribution of these two taxa occurs remains unclear.

Distribution. East Asia (East and South China, including Taiwan), Indochina (Thailand, Cambodia, Vietnam), Malesia (Malaysia) [16,23,27,32,40,43]. The species is hitherto newly reported for Vietnam.

Ecology. Horikawa [43] (p. 196) briefly described the habitat as “on humus”, while Kitagawa [16] (p. 262) indicates “tree trunk in moist evergreen forest”. Our specimens are quite corresponding to the both above and were collected from humus, decaying wood and small wood debris in evergreen tropical forest with substantial participation of *Dacrydium elatum* at the elevation 700–1030 m a.s.l.

Specimens examined: THAILAND. Loey, Mt. Phu Luang, ca. 1500 m a.s.l., on tree trunk in moist evergreen forest, 12 March 2022, M. Tagawa & N. Kitagawa, T1679, isotype of *Bazzania semiopaca*. N. Kitag., NICH-279771 (NICH!); VIETNAM. Central Highlands, Gia Lai Province, Kbang District (14.49050300° N 108.56661000° E), 1030 m a.s.l., evergreen tropical forest with substantial share *Dacrydium elatum*, on small wood debris, on fragments of wood and bark, 12 March 2022, E.V. Kushnevskaya, DAK6 Melky debris kushn4 (VBGI); CAMBODIA. Koh Kong Province, Phumi Chréav (11.80528° N 103.51083° E), 700 m a.s.l., broadleaved evergreen forest on slope, decaying wood, 22 December 2011, V.A. Bakalin, Cam-81–33–11 (VBGI).

*Bazzania appendiculata* (Mitt.) S.Hatt., Fl. E. Himalaya: 505, 1966 ssp. *appendiculata*

Descriptions: [27,31].

**Figure 6:**
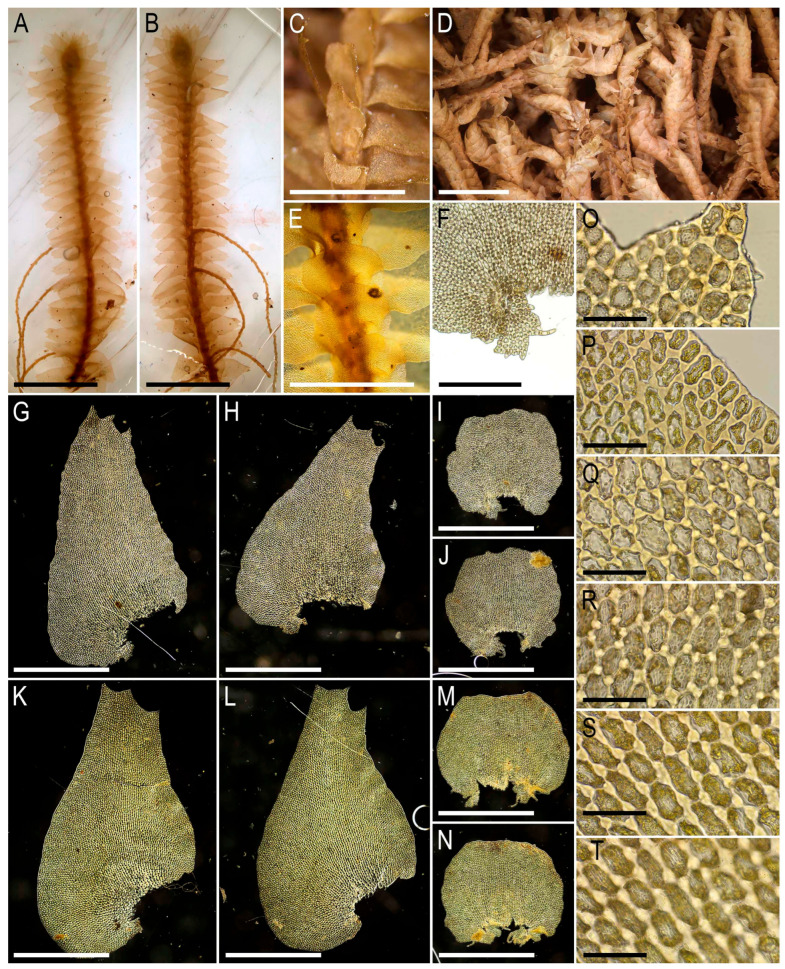
*Bazzania appendiculata* (Mitt.) S.Hatt.: (**A**) shoots with ventral scale-like leaved ‘flagella’, dorsal view; (**B**) shoots with ventral scale-like leaved ‘flagella’, ventral view; (**C**) underleaves on a part of a shoot, latero-ventral in three-quarter view (in dry condition), fragment; (**D**) part of mat in dry condition; (**E**) underleaves on a part of a shoot (in wet condition under the slide); (**F**) appendage at the underleaf base; (**G**,**H**,**K**,**L**) leaves; (**I**,**J**,**M**,**N**) underleaves; (**O**) leaf lobe cells, verrucose leaf cuticle is shown; (**P**) leaf margin cells; (**Q**–**T**) midleaf cells; (**R**,**T**) verrucose leaf cuticle of midleaf area. (**G**–**J**,**K**–**N**) are photographed with dark field option. Scales: 5 mm for (**A**,**B**); 4 mm for (**D**); 2 mm for (**C**,**E**); 1 mm for (**G**–**N**); 300 µm for (**F**); 50 µm for (**O**–**T**). (**C**,**K**–**N**,**P**,**S**,**T**) from eskov5 (VBGI, HN); (**A**,**B**,**D**–**J**,**O**,**Q**,**R**) from V-2-6-22 (VBGI).

Illustrations: [27] (Figure 6); [31] (Figures 1L–R and 4).

Illustrations in the present paper: Figure 6.

Comment. The species was well described in the sources cited above, including original description. It possesses the following characteristic features:

- Strongly pachydemous leaf and underleaf cells;
- Papillose cuticle of leaves and underleaves;
- Obliquely ovate to ovate-triangular leaves that are distinctly three-lobed with slightly diverging lobes;
- Subquadrate–suborbicular underleaves with margin entire to crispate;
- Well-developed and distinctly toothed underleaf appendages.

The most morphologically similar taxon is *Bazzania asymmetrica* (Steph.) N. Kitag., whose distribution stretches from China to Malesia and then Melanesia [23], where *B. appendiculata* is generally broadly Sino-Himalayan although readily spreading eastward. Therefore, the occurrence of *Bazzania asymmetrica* is not impossible in Indochina. *Bazzania appendiculata* is, however, distinct from the latter due to its distinctly verrucose–papillose leaf cuticle (versus cuticle smooth) and underleaves entire to crispate along the margin (versus crispate to lobed and sometimes sparsely dentate underleaf margin in *B. asymmetrica*). In addition, although the leaf cell trigones are large in both taxa, the size of them in *B. asymmetrica* is nevertheless much larger and causes a somewhat vermicular appearance of the midleaf cells, which are additionally elongated more prominently than in *B. appendiculata*.

Distribution. South Asia (Bangladesh, India), East Asia (South and West China, Nepal, Bhutan), Indochina (Thailand, Myanmar, Cambodia—new record, Vietnam—new record), Malaysia [23,27].

Ecology. Cheah et al. [23] identify the habitat as tree trunks and ground at an elevation around 1500 m. The occurrences in Cambodia and Vietnam are essentially the same. The species is growing as an epiphyte or occurs in epilithic habitats in tropical or mountain subtropical (North Vietnam) evergreen forests at elevations of 1000–1500 m a.s.l.

Specimens examined: VIETNAM. Northwest Vietnam, Điện Biên Province, Mường Nhé District, Sín Thầu Commune, Mường Nhé Nature Reserve, Pờ Nhù Khò Mt. (22.37348° N 102.19241° E), 1501 m a.s.l., subapical part of unnamed mountain covered with evergreen tropical forest on NW-facing slope, open mesic cliff, 7 April 2022, V.A. Bakalin & M.H. Nguyễn, V-2-6-22 (VBGI, HN); CAMBODIA. Kampot Province, Preah Monivong (Phnom Bokor) Natonal Park (10.635775° N 104.006254° E), 1040 m a.s.l., mossy forest near the cliffs of the plateau near Bokor Mt., epiphyte, 24 June 2022, A.K. Eskov & N.G. Prilepsky, eskov5, eskov10 (VBGI); *ibid.* mossy forest, epiphyte 27 June 2022, A.K. Eskov & N.G. Prilepsky, eskov66 (VBGI).

*Bazzania appendiculata* subsp. *cambodiana* Bakalin, S.S. Choi, Klimova subsp. nov.

Description. Plants brown to yellowish brown, with leaves strongly turned ventrally when dry and sometimes clasped in ventral side, robust, 30–40 mm long and 3–4 mm wide. Stem 300–400 µm wide, sparsely branched by *Frullania*-type branching, with more or less common ventral scale-like leaved ‘flagella’. Rhizoids seen only in distal part of ventral flagella, colorless to light brownish, originating from stem in the areas adjacent to modified leaves and underleaves, obliquely spreading. Leaves imbricate, convex to ridged (thus concave-canaliculate if to view from the ventral side), covering 1/3–1/2 of the above situated leaf, obliquely oriented, laterally suberect spreading, in ventral side with obscure appendages with teeth or without them, in dorsal side arcuately inserted, with small appendage, with teeth or without them, when flattened in the slide obliquely ovate, 1500–1800 × 1200–1400 µm, distinctly 3–lobed, with diverging lobes, not vittate. Midleaf cells oblong, (20–)25–35(–40) × 15–20 µm, thin-walled, with large, bulging trigones, cuticle papillose; cells along margin 15–20 µm, thin- to thick-walled, external wall mostly thickened, trigones large, sometimes confluent, triangle to concave when adjacent to external wall and nodulose inward, cuticle papillose-verrucose. Underleaves overlapping 1/3 of the above situated underleaf, obliquely spreading, concave-canaliculate, when flattened in the slide suborbicular, with large toothed appendage near bases, 700–900 × 900–1000 mm, connate with the leaf in one side, with long persistent, brown colored slime papillae along underleaf margin. Perianth on reduced ventral branch appearing in underleaf sinus, ovate-fusiform, 4300 × 2000 µm [mouth broken and its features are not seen], with 3 pairs of bracts, bracts irregularly lobed and dentate-ciliate, innermost 2700 µm long, middle—1700 µm long, outermost—600 µm long, imbricate.

Holotype: CAMBODIA. Kampot Province, Bokor National Park, below the Popokvil waterfall (10.66236° N 104.04844° E), 872 m a.s.l., near waterfall, 12 January 2012, S.S. Choi, C2317 (JNU, dupl. in VBGI).

**Figure 7:**
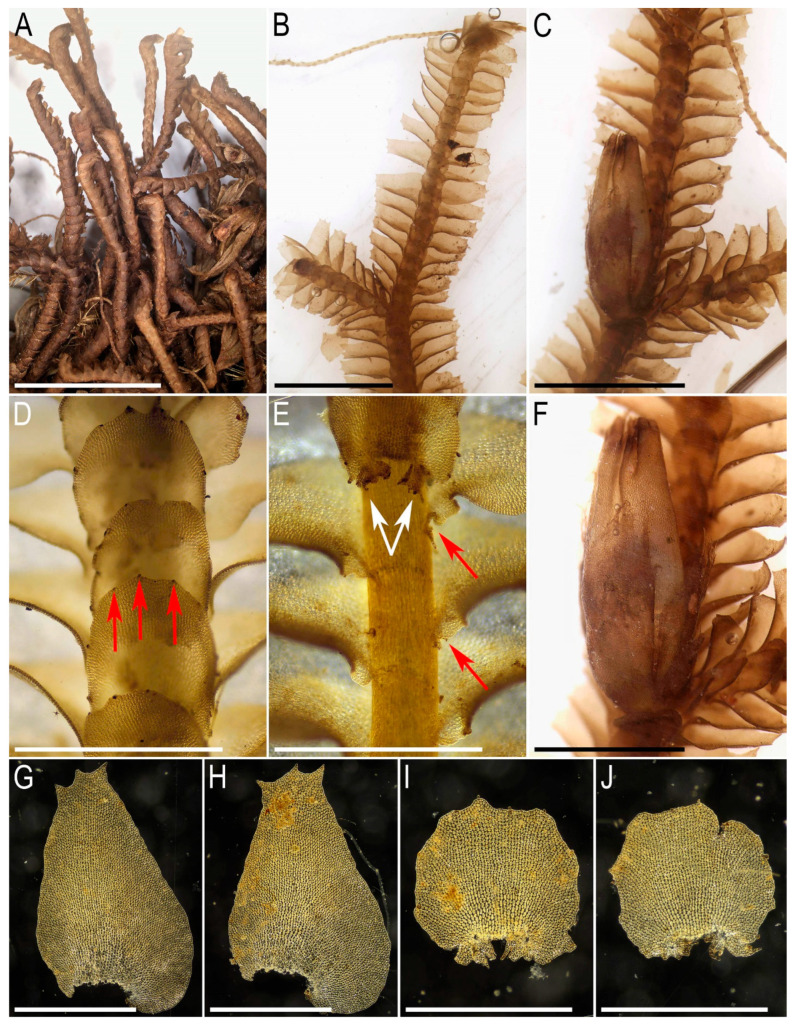
*Bazzania appendiculata* ssp. *cambodiana* Bakalin, S.S. Choi, Klimova subsp. nov.: (**A**) part of mat in dry condition; (**B**) part of shoot, ventral view; (**C**) part of shoot bearing mature perianth, ventral view; (**D**) underleaves with brown colored slime papillae along margin (indicated by red arrows) on a part of a shoot; (**E**) part of a shoot in ventral view: underleaf with large toothed appendages near base (indicated by white arrows), leaves with obscure appendages with teeth or without them (indicated by red arrows); (**F**) mature perianth on a shoot; (**G**,**H**) leaves; (**I**,**J**) underleaves. (**G**–**J**) are photographed with dark field option. Scales: 10 mm for (**A**); 3 mm for (**B**,**C**); 2 mm for (**F**); 1 mm for (**D**,**E**,**G**–**J**). All from C2317 (JNU, dupl. in VBGI).

**Figure 8:**
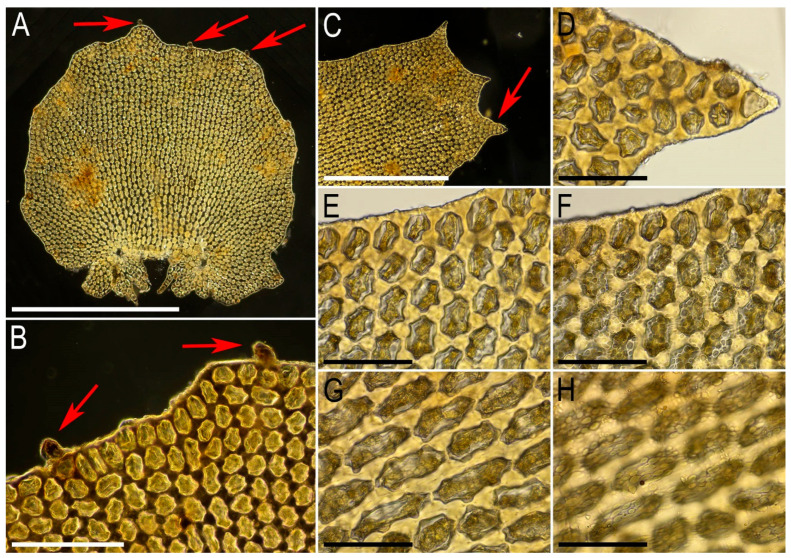
*Bazzania appendiculata* ssp. *cambodiana* Bakalin, S.S. Choi, Klimova subsp. nov.: (**A**) underleaf with slime papillae along margin pointed by red arrows; (**B**) underleaf apex with slime papillae along margin pointed by red arrows; (**C**) leaf apex, lobe pointed by red arrow is shown in (**D**); (**D**) lobe apex cells; (**E**,**F**) leaf margin cells; (**G**,**H**) mifleaf cells; (**F**) papillose–verrucose cuticle in leaf margin; (**H**) papillose cuticle in midleaf area. (**A**–**C**) are photographed with dark field option. Scales: 1 mm for (**A**); 500 µm for (**C**); 100 µm for (**B**,**D**–**H**). All from C2317 (JNU, dupl. in VBGI).

Illustrations in the present paper: Figure 7 and Figure 8.

Comment. The subspecies is characterized by the same basic features as listed above for ssp. *appendiculata*. However, ssp. *cambodiana* is strikingly different from the type of subspecies (ssp. *appendiculata*) due to its brown color (versus yellowish brownish in ssp. *appendiculata*), as well as its long persistent deeper-colored slime papillae in underleaf apex (as commonly observed in otherwise different *B. himalayana* (Mitt.) Schiffn.); wider (=more shortly ovate) leaves—although this feature is very variable in ssp. *appendiculata*; shorter (tending to be round) papillae in midleaf cell surface. Two subspecies may be sympatrically distributed. At least the true ssp. *appendiculata* also occurs in Cambodia (see above). The taxon status needs verification.

The issue of the perimeters of morphological variability of the *Lepidoziaceae* taxa, and *Bazzania* in particular, is still far from being correctly understood. The known sequences of various loci for most species are based on one or two original specimens, often collected far from the type localities [4]. Therefore, the interpretation of the available data can hardly extend much beyond a discussion of the genetic differences in the particular specimens and move on to the level of distinguishing species, since there is no general criteria. The *Bazzania* revision for New Caledonia [42] provides illustrations of *B. tridens* (p. 149), which differs strikingly from the type material of the species [31] in the nature of the leaf margin—coarsely serrulate, as opposed to completely entire or nearly so. Nevertheless, it is hardly possible to immediately reject the correctness of the species identification in New Caledonia without conducting an integrative comparison. However, if such morphological variability is genetically confirmed for *B. tridens*, it will force a revision of the morphological concepts of other species. Returning to the *B. appendiculata* ssp. *cambodiana* described here, it can be said that the nature of its morphological differences, apart from the ecology, likely corresponds to the rank of species. However, the lack of clear data on the correlation of genetic structure with morphological variability precludes a definitive answer to this question. Whether the cited morphological differences are environmentally induced can also be determined through molecular genetic studies. Unfortunately, *Bazzania* are difficult to study molecularly (in particular, the amplification of the most popular loci), and further targeted research, including the search for new molecular analysis methods, is necessary to resolve this issue.

Ecology. The subspecies is found on the open presumable mesic cliffs surrounded by dense tropical evergreen rather secondary forest at the elevation 872 m a.s.l.

*Bazzania callida* (Sande Lac. ex Steph.) Abeyw., Ceylon J. Sci., Biol. Sci. 2 (1): 45, 1959

Descriptions: [44].

Illustrations: [44] (Plate 1, Figure 2).

Description. Plants prostrate, brownish yellowish to brownish when dry, somewhat glistening, in loose patches, 10–30(–60) mm long and 1.8–2.3 mm wide, sparsely branched (*Frullania*-type). Rhizoids virtually absent in normally leaved shoots, but sparsely present in distal parts of the rater abundant ventral scale-like leaved ‘flagella’, where originating from the stem in the areas just below the modified leaves and underleaves. Leaves contiguous to shortly imbricate (overlapping to 1/5 of the above situated leaf in the base), obliquely inserted, slightly convex-ridged, subhorizontally oriented, suberect spreading (both in dry and wet conditions), when flattened in the slide obliquely ovate, 800–1000 × 500–700 µm in the middle of the lower half of the leaf with the large-celled zone, very shortly, distinctly to unclearly 3–lobed. Cells in the midleaf oblong, in pseudovittate area 30–42 × 17–20(–25) µm, thin-walled to walls slightly thickened, trigones moderate to large in size, concave to slightly convex, cuticle smooth; cells aside pseudovittate area nearly subisodiametric to shortly oblong, 15–22(–25) µm in diameter to 20–23 × 15–20 µm, with thin to somewhat thickened walls, trigones moderate to large in size, concave, somewhat confluent, cuticle smooth; cells along margin in leaf dorsal side, (10–)12(–15) µm, subquadrate, with thick walls and small trigones, cuticle verrucose; near leaf apex margin cells 14–17 × 12–15 µm, with walls thick, trigones small, concave, cuticle verrucose (verrucae 1.5–2.0 µm in diameter). Underleaves tightly appressed to the stem, hyaline except area very near the base or chlorophyllose up to 1/4 of the underleaf length, subquadrate to obtrapezoidal, shortly dentate along margin, 300–500 × 300–450 µm, underleaf cuticle smooth.

**Figure 9:**
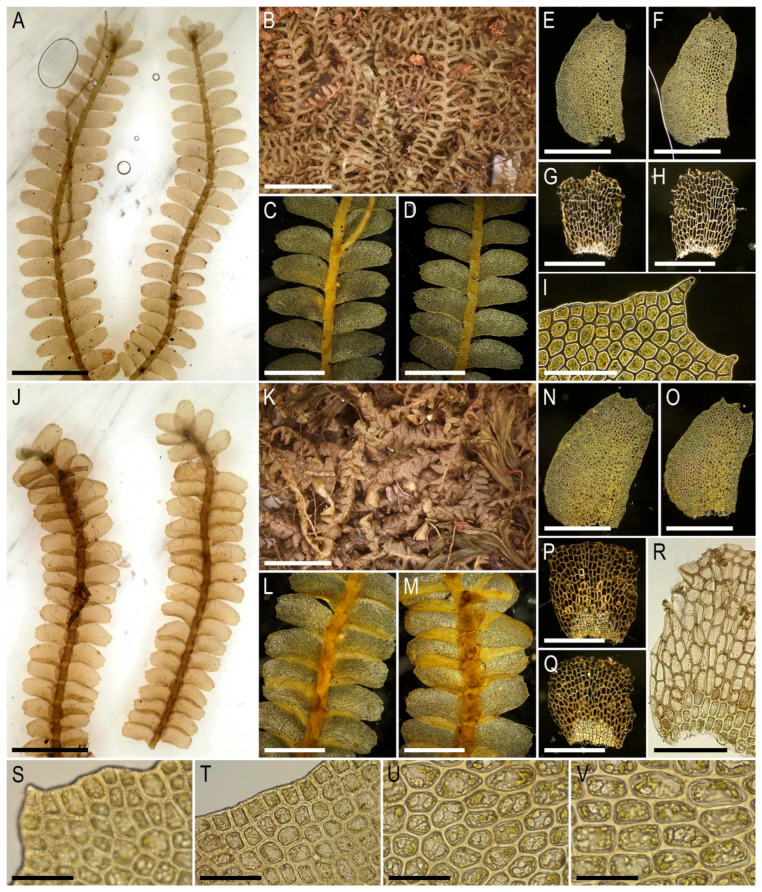
*Bazzania callida* (Sande Lac. ex Steph.) Abeyw.: (**A**,**J**) shoots, the left one in dorsal view, the right one in ventral view; (**B**,**K**) part of mat in dry condition; (**C**,**L**) part of shoot, dorsal view; (**D**,**M**) part of shoot, ventral view; (**E**,**F**,**N**,**O**) leaves; (**G**,**H**,**P**,**Q**) underleaves; (**I**) leaf apex cells; (**R**) part of underleaf; (**S**) verrucose cuticle in leaf apex; (**T**) leaf margin cells; (**U**) cells in pseudovitta area; (**V**) midleaf cells in pseudovitta area. (**C**–**I**,**L**–**Q**) are photographed with dark field option. Scales: 3 mm for (**B**,**K**); 2 mm for (**A**,**J**); 1 mm for (**C**,**D**,**L**,**M**); 500 µm for (**E**,**F**,**N**,**O**); 300 µm for (**G**,**H**,**P**,**Q**); 100 µm for (**I**,**R**); 50 µm for (**S**–**V**). (**A**–**I**) from Cam-83-51-11, (**J**–**V**) from Cam-88-27-11 (VBGI).

**Figure 10:**
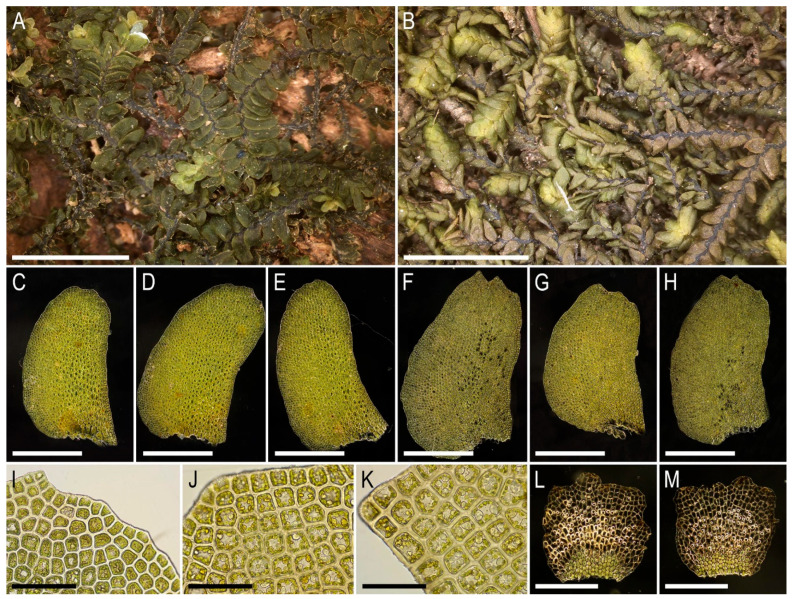
*Bazzania callida* (Sande Lac. ex Steph.) Abeyw.: (**A**,**B**) part of mat in dry condition; (**C**–**H**) leaves; (**I**–**K**) leaf apex cells; (**L**,**M**) underleaves. (**C**–**H**,**L**,**M**) are photographed with dark field option. Scales: 6 mm for (**A**,**B**); 500 µm for (**C**–**H**); 300 µm for (**L**,**M**); 50 µm for (**I**–**K**). (**A**,**C**–**E**,**I**) from KON1 Derevo1komel kushn5, (**B**,**F**–**H**,**J**–**M**) from L-6-11-25 (VBGI).

Illustrations in the present paper can be found in Figure 9 and Figure 10.

Distribution. South Asia (0Sri Lanka), Indochina (Thailand, Cambodia, Laos—new report, Vietnam—new report).

Ecology. Kitagawa [16] (p. 262) describes habitat in Thailand as “350–600 m, on soil in dry deciduous forest”. In Vietnam, the predominant habitats are tree trunk bases, and sometimes decaying wood in evergreen forests at 1000 m a.s.l. In Cambodia the species occurs on stone sides in dry evergreen forests at elevations 360–700 m a.s.l. The ecology in Laos is similar to above, where it occurs on open dry cliffs near stream at the elevation 354 m a.s.l. The species is probably the most xerophytic and the taxon occurring in the lowest elevation in the genus occurring in Indochina.

Specimen examined: CAMBODIA. Koh Kong Province, Phumi Chréav (11.81556° N 103.47611° E), 700 m a.s.l., broadleaved evergreen forest on slope, stone side, 25 December 2011, V.A. Bakalin, Cam-88–27–11 (VBGI); VIETNAM. North Central Coast, Quang Tri Province, Bố Trạch District, Phú Định Commune, northern part of Annamite Range (Trường Sơn Range), Phong Nha–Kẻ Bàng National Park, surroundings of U Bò Mountain Ranger Station (17.46842° N 106.37622° E), 896 m a.s.l., saddle on a ridge covered by low tropical montane evergreen forest with major broadleaf species and palms (*Livistona*, *Lanonia*, etc.), *Pandanus* and dwarf tree fern in understory, partly shaded moist stone, 26 April 2025, V.A. Bakalin, K.G. Klimova & M.H. Nguyễn, V-22-13-25 (VBGI, HN); *ibid.* the border of buffer zone of Phong Nha–Kẻ Bàng National Park, Hồ Chí Minh Trail (17.40308° N 106.47794° E), 69 m a.s.l., secondary tropical evergreen forest along the mountain road, open mesic cliff on the road cut, 25 April 2025, V.A. Bakalin, K.G. Klimova & M.H. Nguyễn, V-17-5-25 (VBGI, HN).

Comment. The species experience some morphological variation; the following deviations were observed:

Specimen Cam-83–51–11 differs from above in distant to contiguous leaves and rather oblong underleaves (0.45 × 0.3 mm).

Specimen KON1 Derevo1komel kushn5 characterized by bright green coloration (probably due to shady conditions and more fresh material) and only unclearly lobed leaves—in that feature, the plants are even more similar to the type than in the description above.

Specimen L-6–11–24 characterized by bright green coloration and plants size larger, reaching 2.1–2.8 mm wide. Correspondingly, leaves are larger, up to 1.4 × 0.8 mm, larger leaves are more clearly lobed, although the great variation observed in this feature within the mat; underleaves somewhat lobed, subquadrate to 0.5 × 0.5 mm. Moreover, it seems all the specimens from Laos are more brightly colored and larger, and leaves are more clearly trilobed. The status of those plants may require verification.

Specimens examined for deviations: VIETNAM. Central Highlands, Gia Lai Province, Kbang District (14.50000100° N 108.54515600° E), 1049 m a.s.l., evergreen tropical forest, on bark at base alive tree trunk, 7 March 2022, E.V. Kushnevskaya, KON1 Derevo1komel kushn5 (VBGI); CAMBODIA. Koh Kong Province, Thma Bang Village area (11.66194° N 103.39861° E), 360 m a.s.l., broadleaved evergreen forest on slope to waterfall, more or less dry cliff near waterfall (sometimes sprayed area), 23 December 2011, V.A. Bakalin, Cam-83–51–11 (VBGI); LAOS. Bolikhamsai Province, Thaphabath Commune (18.43588° N 102.95078° E), 354 m a.s.l., evergreen secondary tropical forest along sluggishly flowing stream in wide valley, open dry cliff near the stream, 8 May 2024, V.A. Bakalin, L-6–11–24 (VBGI).

*Bazzania commutata* (Lindenb. et Gottsche) Schiffn., Consp. Hepat. Arch. Ind.: 149, 1898

Descriptions: [45].

Description. Plants large, robust, creeping to loosely ascending, yellowish with brownish tint in lower part, freely branched (branching *Frullania*-type), ventral ‘flagella’ with scale-like leaves abundant, 30–50 mm long and 3–4 mm wide. Rhizoids absent in leaves shoots, very sparse in terminal portions of ventral ‘flagella’, where, although, in the vast majority of cases totally absent. Leaves obliquely inserted and oriented suberect spreading, dorsally insertion line arcuate, ridged, imbricate, covering 2/3–3/4 of the above situated leaf, when flattened in the slide obliquely ovate-falcate, from very wide base to the narrow, distinctly trilobed apex, antical side strongly arcuate, postical—almost straight to slightly arcuate, 1500–1900 × 1200–1400 µm, crenulate-denticulate in lower part of the leaf, and denticulate to dentate in upper 1/3, therefore lobes with numerous accessory teeth. Midleaf cells although longer than in the margin, but never form pseudo-vitta area, oblong, 40–50(–75) × 25–30(–35) µm, thin-walled, with large, nodulose trigones with visible middle lamina, cuticle virtually smooth. Cells along margin in antical side (20–)25–30 µm, thin-walled, with strongly thickened external wall, where many cell walls continue thickenings to form short denticulation, cuticle verrucose just along the margin. Cells in apical part of the leaf 25–45 × 25–35 µm, thin-walled, with nodulose trigones, with visible middle lamina, cuticle distinctly papillose-verrucose along margin, inward soon become nearly smooth. Underleaves concave, connate with leaves in one or both sides, with recurved apex, obliquely to suberect spreading, contiguous, when flattened in the side transversely ellipsoidal to subquadrate, with small auricles near the base, apex shortly to indistinctly 3–4–lobed, with rounded lobes, 600–900 × 1000–1200 µm, in apical part densely denticulate, in lateral sides only crenulate.

**Figure 11:**
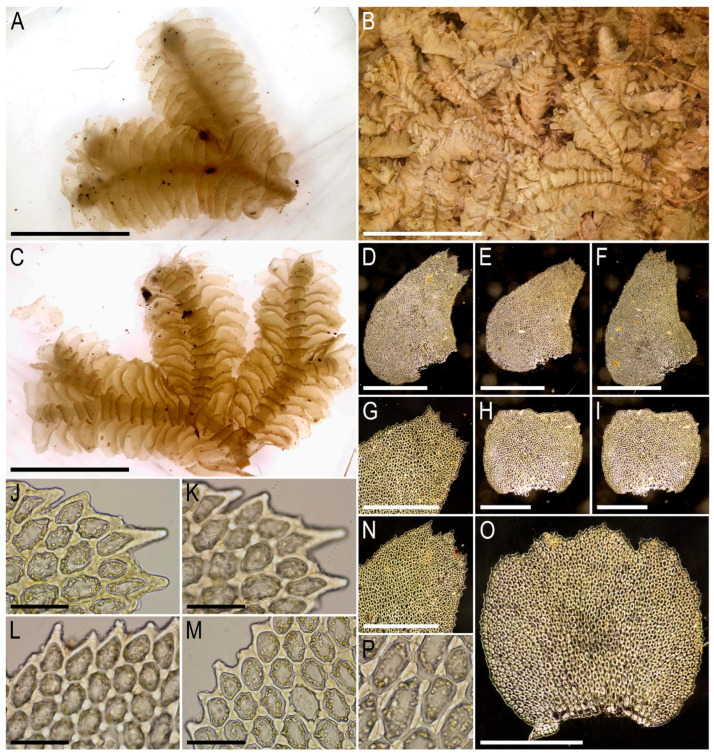
*Bazzania commutata* (Lindenb. et Gottsche) Schiffn.: (**A**) shoots, dorsal view; (**B**) part of mat in dry condition; (**C**) shoots, ventral view; (**D**–**F**) leaves; (**G**,**N**) leaf apex; (**H**,**I**,**O**) underleaves; (**J**,**K**) cells of leaf lobe apex; (**K**) papillose–verrucose cuticle in lobe apex area; (**L**,**M**) leaf margin cells; (**L**) verrucose cuticle in leaf margin area; (**P**) midleaf cells. (**D**–**I**,**N**,**O**) are photographed with dark field option. Scales: 6 mm for (**B**); 4 mm for (**A**,**C**); 1 mm for (**D**–**F**); 500 µm for (**G**–**I**,**N**,**O**); 50 µm for (**J**–**M**,**P**). All from Eskov Pn-9 (VBGI).

Illustrations in the present paper can be found in Figure 11.

Comment. *Bazzania commutata* is well characterized by the following features:

1. Three–four-lobed leaves with numerous teeth and denticulations descending from the leaf apex in both lateral sides of the leaf;
2. Recurved subquadrate to transversely ellipsoidal underleaves, indistinctly lobed and denticulate throughout;
3. Leaf with wide base and strongly narrowing to the apex;
4. Prominently nodulose trigones in leaf cells with visible middle lamina.

Kitagawa [19] mentions the similarity of the species and *Bazzania confertifolia* (Steph.) Herzog, from which *B. commutata* differs in leaf lobation (leaves short but rather distinctly lobed versus unlobed in *B. confertifolia*), and subquadrate underleaves (versus reniform). Also the leaf apex seems to be more wide in *B. commutata* than in *B. confertifolia*. Kitagawa [19] (p. 80) wrote that “intermediate forms” were observed between two species. Therefore, further study is required to resolve this issue.

Among the regional representatives of the genus, the species also resembles: (1) *Bazzania erosa*, from which is distinguished by the absence of a hyaline border along the underleaf margin; (2) *Bazzania indica* (Gottsche et Lindenb.) Trevis. (the name may be misleading, actually the species was described from Singapore), from which *B. commutata* differs in dentate-denticulate leaf margin with lobes bearing numerous accessory teeth and densely dentate-denticulate underleaf margin. Illustrations of *B. indica* are published by Bakalin, Maltseva [31] and Pócs et al. [29].

Distribution. Indochina (Vietnam—new report); Malesia (Malaysia, Indonesia: Java Island, Moluccas) [23,40,45,46]. Furthermore, Stephani [45] points out that very similar plants were observed in the Duthie collection from the Himalayas, but the material there is very scant, precluding further estimations.

Ecology. The species occurs in Vietnam at an elevation above 1800 m a.s.l. in evergreen forests, as epiphytes in the complex moss mat over tree trunks. We could not find any other data on the species ecology in the available literature.

Specimens examined: VIETNAM. Central Highlands, Lâm Đồng Province, Lạc Dương District, southern part of Annamite Range (Trường Sơn Range), Lâm Viên Plateau, Bidoup Núi Bà National Park, Hòn Giao Mt. (12.19° N 108.710556° E), 1861 m a.s.l., mossy mats over tree trunks, 23 November 2018, A.K. Eskov & N.G. Prilepsky, Eskov Pn-9 (VBGI).

*Bazzania erosa* (Reinw., Blume et Nees) Trevis., Mem. Reale Ist. Lombardo Sci. (Ser. 3), C. Sci. Mat. 4 (13): 415, 1877.

Descriptions: [18,41]. Note, the description in [47] corresponds to *Bazzania longicaulis* (cf. [18]).

Illustrations: [41] (Figure 11); [9] (Plate 16, Figures 1–10), [18] (Figures 1–13). Note, the description in [47] (Plate XVI) corresponds to *Bazzania longicaulis* (cf. [18]).

Description. Plants rigid, robust, in loose patches, brownish green to yellowish brownish in the herbarium, sparsely branched (branching *Frullania*-type) and with common to numerous ventral ‘flagella’ with scale-like leaves, 30–70 mm long and 3.2–5.0 mm wide. Rhizoids absent in normally leaves shoots, but present in the terminal portions of ventral ‘flagella’ where from the stem in the areas adjacent both to the bases of the modified leaves and underleaves, obliquely to erect spreading, colorless, shorter 1 mm in length. Leaves obliquely inserted and oriented, suberect spreading, slightly ridged, dorsally insertion line arcuate, imbricate, covering 1/2–2/3 of the above situated leaf in its base (apical part is not overlapped or only slightly so), when flattened in the slide obliquely ovate to narrowly obliquely ovate and rarely somewhat falcate, shortly, but in the vast majority of the cases, distinctly 3-lobed with numerous accessory teeth and also dentate along leaf margin in apical 1/3–1/4 of the leaf, below commonly denticulate, on general outline 1800–2200 × 1200–1400 µm. Midleaf cells oblong, 30–45(–50) × (18–)20–25 µm, thin-walled, trigones large, nodulose, with visible middle lamina, cuticle more or less smooth. Cells along margin 20–25(–30) µm, external wall variously thickened and producing denticulations, radial walls unequally thickened, tangential (innermost to the midleaf) thin, trigones large, nodulose to concave, sometimes confluent, cuticle verrucose. Cells in the apical part of the leaf 25–35 × 15–25(–30) µm, oblong to subisodiametric, thin-walled, trigones large to moderate in size, nodulose, cuticle distinctly papillose. Underleaves concave (if to look from ventral side), obliquely to narrowly so spreading, with apical part sometimes erect spreading, never reflexed or recurved, connate in the both sides with the leaves (rarely in one side only), shortly auriculate near the base, when flattened in the slide more or less reniform to transversely ellipsoidal, 500–700 × 700–1000 µm, along margin with 1–3 rows of cells discolored (not chlorophyllose) and thin-walled. Mature perianth fusiform, 3500–4000 × 1200 µm, basically 3–plicate in upper half, sometimes with additional folds, with three pairs of bracts. Outer bracts ovate, 400–500 µm long, bracteole narrowly ovate, with acuminate apex; middle bracts ovate, 1000–1200 µm long, with apex laciniate, bracteole ovate ca. 600 µm long; inner bracts ovate, 1700–1800 µm long, with laciniate apex, bracteole 1200 µm long, with laciniate apex.

**Figure 12:**
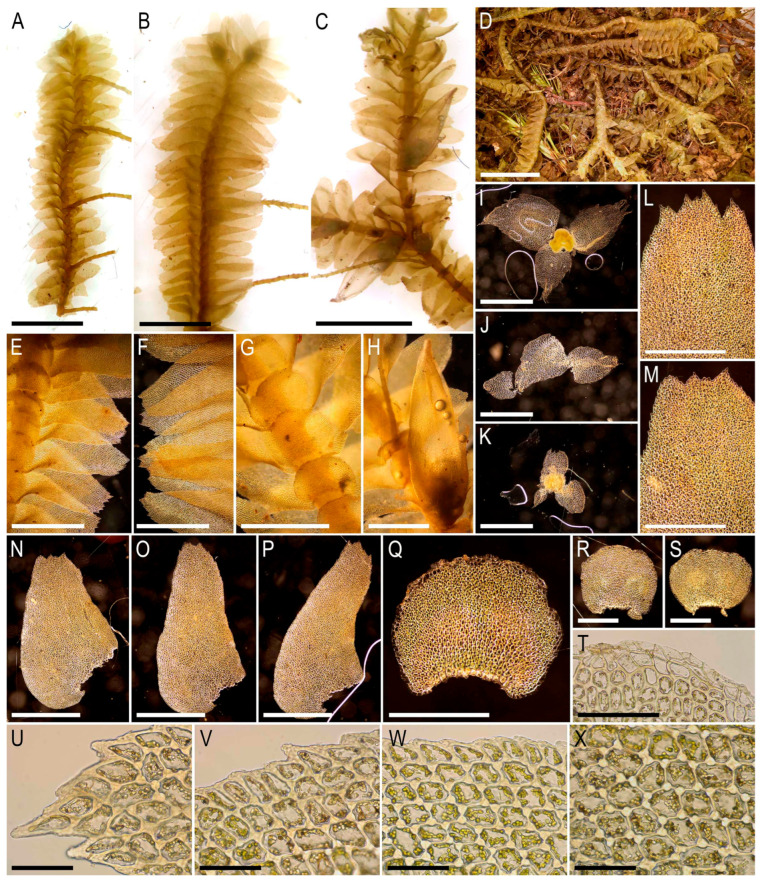
*Bazzania erosa* (Reinw., Blume et Nees) Trevis.: (**A**) shoot with ventral scale-like leaved ‘flagella’, dorsal view; (**B**) shoot with ventral scale-like leaved ‘flagella’, ventral view; (**C**) part of shoot with mature perianths, ventral view; (**D**) part of mat in dry condition; (**E**) leaves on a shoot, ventral view; (**F**) leaves on a shoot, dorsal view; (**G**) underleaves on a shoot, ventral view; (**H**) mature perianth on a shoot, ventral view; (**I**) inner bracts; (**J**) middle bracts; (**K**) outer bracts; (**N**–**P**) leaves; (**Q**–**S**) underleaves; (**T**) rows of discolored (not chlorophyllose) thin-walled cells in underleaf apex margin; (**U**) lobe apex cells; (**V**,**W**) leaf margin cells; (**X**) midleaf cells. (**E**–**S**) are photographed with dark field option. Scales: 6 mm for (**D**); 4 mm for (**C**); 3 mm for (**A**,**B**); 1 mm for (**I**–**K**,**N**–**P**); 500 µm for (**L**,**M**,**Q**–**S**); 100 µm for (**T**); 50 µm for (**U**–**X**). (**C**,**G**–**K**) from Eskov 57, (**A**,**B**,**D**,**E**,**F**,**L**–**X**) from Eskov 71 (VBGI).

Illustrations in the present paper can be found in Figure 12.

Comment. Cheah et al. [23] propose synonymy of the *B. erosa* and *B. serrulatoides* Horik., which is difficult to disagree with. However, the description and drawings by Horikawa [43] suggest crispate underleaf margin (Figure 33 in l.c.). Therefore, this issue requires further study. The data on the occurrence of *B. serrulatoides* in Indonesia, Thailand and China [27] may be based on *B. erosa*. Differences from the morphologically most similar species are given in detail by Cheah et al. [23] as well as Kitagawa [18]. In addition, both studies point out the significant variability of this widespread species in Southeast Asia. Some problems may arise in distinguishing *B. erosa* from *B. spiralis* (Reinw., Blume et Nees) Meijer. The last of the differences cited by Kitagawa [19] (p. 75) is the distanced underleaves, which are “strongly deflexed at the apex” in *B. spiralis*. However, in our *B. erosa* plants, the underleaves may not overlap but rather be contiguous; however, they are never strongly deflexed and crispate. Pócs et al. [30] has reported *B. serrulatoides* from Vietnam (Bidoup-Núi Bà National Park). We were not able to understand whether they maintained the differences between *B. serrulatoides* and *B. erosa* at the species level, because the authors did not mention the latter in the discussion.

Distribution. Stretching area from South Asia (Sri Lanka), East Asia (China), Indochina (Thailand, Cambodia, Vietnam), Malesia (Philippines, Malaysia, Indonesia) to Melanesia, Australasia and Oceania [18,23,27,30,40,41], in [27,30] described under *B. serrulatoides*.

Ecology. Across its large area the species shows uniform ecology growing on tree trunks, decaying wood and ground in humid evergreen montane forests at elevation above 1400 m [23,25]. The ecology in Cambodia is similar, the species was found as epiphyte over tree trunk near the ridge slightly above 1000 m a.s.l.

Specimens examined: CAMBODIA. Kampot Province, Preah Monivong (Phnom Bokor) Natonal Park (10.635775° N, 104.006254° E), 1050 m a.s.l., mossy forest on the cliff, epiphyte, 27 June 2022, A.K. Eskov & N.G. Prilepsky, Eskov71, eskov57, eskov60 (VBGI); ibid. (10.638712° N 104.004770° E), 1050 m a.s.l., mossy forest near the cliff of the plateau, epiphyte, 26 June 2022, A.K. Eskov & N.G. Prilepsky, eskov20 (VBGI).

*Bazzania fleischeri* (Steph.) Abeyw., Ceylon J. Sci., Biol. Sci. 2 (1): 45, 1959

Descriptions: [27].

**Figure 13:**
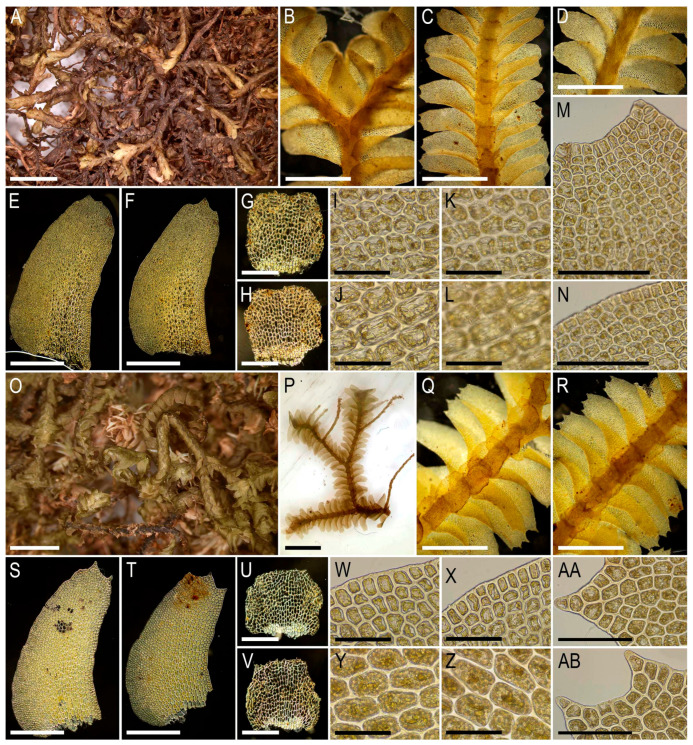
*Bazzania* cf. *fleischeri* (Steph.) Abeyw. (*Bazzania* cf. *fleischeri* 1, Cam-89–10–11): (**A**) part of mat; (**B**,**D**) shoots, dorsal view; (**C**) shoots, ventral view; (**E**,**F**) leaves; (**G**,**H**) underleaves; (**I**,**K**) cells in the midleaf aside of pseudovitta; (**K**) unclearly papillose cuticle in the midleaf aside of pseudovitta; (**J**,**L**) pseudovitta cells in the midleaf; (**L**) papillose cuticle of pseudovitta area in the midleaf; (**M**) cells of leaf apex; (**N**) cells of leaf margin. *Bazzania* cf. *fleischeri* (Steph.) Abeyw. (*Bazzania* cf. *fleischeri* 2, DAK1 valezh1 kushn6): (**O**) part of mat; (**P**) shoot, ventral view; (**Q**,**R**) shoots, ventral view; (**S**,**T**) leaves; (**U**,**V**) underleaves; (**W**,**X**) cells of the leaf margin; (**Y**,**Z**) cells of the large-celled zone in the midleaf; (**AA**,**AB**) cells of leaf apex. (**B**–**H**,**Q**–**V**) are photographed with dark field option. Scales: 2 mm for (**A**,**O**,**P**); 1 mm for (**B**–**D**,**E**,**F**,**Q**–**T**); 500 µm for (**G**,**H**,**U**,**V**); 100 µm for (**M**,**N**); 50 µm for (**I**–**L**,**W**–**Z**,**AA**,**AB**). (**A**–**N**) from Cam-89–10–11, (**O**–**AB**) from DAK1 valezh1 kushn6 (VBGI).

**Figure 14:**
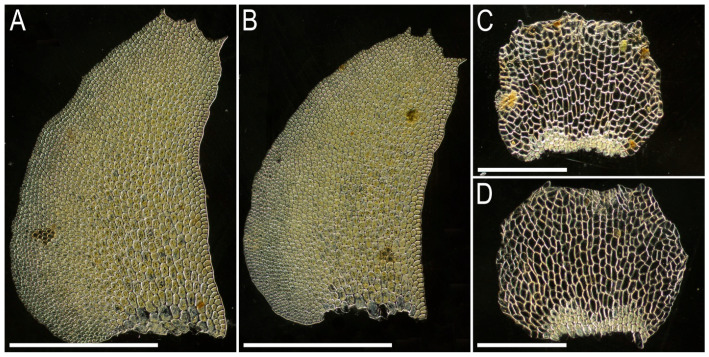
*Bazzania fleischeri* (Steph.) Abeyw.: (**A**,**B**) leaves; (**C**,**D**) underleaves. All are photographed with dark field option. Scales: 500 µm for (**A**,**B**); 300 µm for (**C**,**D**). (**A**–**D**) from DAK9 valezh2 kushn3 (VBGI).

Illustrations: [26] (Figure 7); [27] (Figure 14); [13] (Figures VI: 10–17).

*Bazzania fleischeri*—is a puzzling species for us. We never see the ‘typical’ variant that could be immediately and undoubtedly referred to this taxon. However, we observed some variations which we cannot refer nor to ‘true’ *B. fleischeri* neither to another species and describe the morphology of the plants here under *B.* cf. *fleischeri*. Two main modifications (the actual status needs clarification) may be recognized.

*Bazzania* cf. *fleischeri* 1 (Specimen Cam-89–10–11)

Description. Plants brownish to yellowish brown, merely rigid, in loose patches, 20–30 mm long and 1.8–2.5 mm wide, freely branched (*Frullania*-type), ventral scale-like leaved ‘flagella’ common, commonly branched again. Rhizoids absent in normally leaved shoots, but present in terminal parts of the ventral ‘flagella’ wherefrom the stem in the areas just below of modified leaves and underleaves, pale brownish, obliquely spreading. Leaves obliquely inserted, slightly convex-ridged, subhorizontally oriented, suberect spreading, contiguous to subimbricate, overlapping 1/5–1/3 of the above situated leaf, when flattened in the slide obliquely ovate, somewhat falcate, 1100–1300 × 500–600 µm, only shortly trilobed or unclearly so. Leaves somewhat pseudovittate, pseudovitta cells in the midleaf 25–50 × 20–25 µm, thin-walled, with moderate in size, mostly concave trigones, cuticle papillose (but not verruculose); cells in the midleaf aside of pseudovitta subisodiametric, 12–15(–17) µm, thin-walled with small to rarely moderate in size trigones, cuticle unclearly papillose; cells along dorsal leaf margin 10–12 µm, walls somewhat thickened, trigones small, concave; cells in apical part of leaf along margin 12–15 µm, with walls unequally thickened, trigones small, concave. Underleaves subquadrate, hyaline except area very near the base, 400–500 × 400–500 µm, unclearly crispate along margin, tightly appressed to the stem.

Illustrations in the present paper can be found in Figure 13.

Comment. The plants in the specimen are similar to *B. fleischeri* in their dark coloration with leaves characterized by thick-walled and small cells along the leaf margins and a ‘pseudovittate’ cell organization in the leaf midline. However, the plants in the specimen differ from typical *B. fleischeri* in (1) having very shortly trilobed leaves and (2) having underleaves that are quite narrow but not wider than they are long. In any case, we have not yet been able to find a better name for the plant in the specimen. The plants in the specimen are somewhat similar to *B. tridens*, from which they differs in (1) having very shortly trilobed leaves and (2) having a border of small thick-walled cells along the leaf margin.

*Bazzania* cf. *fleischeri* 2 (Specimens DAK1 valezh1 kushn6, DAK9 valezh2 kushn3)

Description. Plants deep brown green, relatively rigid, 20–40 mm long and 1.8–2.5 mm wide, in loose patches, freely branched (*Frullania*-type). Rhizoids in terminal parts of ventral scale-like leaved ‘flagella’, from the stem, in the areas adjacent to the bases of modified leaves and underleaves. Leaves obliquely inserted, subhorizontally oriented, suberect spreading, slightly ridged, overlapping 1/3–1/2 of above situated leaf near its base, when flattened in the slide obliquely ovate and somewhat falcate, shortly but distinctly (2–)3–lobed, sometimes with fourth lobe or with small solitary accessory teeth, 1000–1300 × 600–800 µm. Large-celled zone in the leaf axis area large and distinct, composed by cells 30–50 × 25–35 µm, walls thin, trigones moderate slightly concave to slightly convex, cuticle smooth. Cells along dorsal margin 7–12 µm, think-walled, external wall thickened, cuticle verrucose very near to the margin, trigones small to moderate in size, concave. Cells in apical area 20–25(30) µm in diameter, very thick-walled, trigones small to moderate in size, concave. Underleaves tightly appressed to the stem, hyaline except area near the base, subquadrate, transversely rectangular and even trapezoidal, 400–500 × 450–600 µm, margin weakly irregularly dentate or loosely crispate.

Illustrations in the present paper can be found in Figure 13 and Figure 14.

Comment 2. Another morphotype similar to *Bazzania fleischeri* is the morphological grading of both *B. albifolia* and *B. tridens*. It differs from typical *B. fleischeri* in that the border is expressed only along the dorsal leaf margin, not the ventral one; the underleaves are nearly as wide as they are long. The leaf apices are generally similar in their thick-walled marginal cells to *B. callida*; however, the cuticle is virtually smooth, being only slightly verrucose at the leaf apex. The distinct small-celled zone in the dorsal side of the leaf and distinct leaf lobation and deeper pigmentation are characteristic for the plants in the specimen. Overall, the plants in the specimen exhibit a combination of (1) thickened walls and somewhat large cells at the leaf apex; (2) a small-celled border along the dorsal leaf margin only; (3) the presence of accessory leaf lobe or few accessory teeth. These characteristics place the plants in an intermediate position between a number of species, including *B. fleischeri*, *B. callida*, *B. tridens*, and *B. intermedia*. Moreover, the mat in the specimen is a mixture of *B.* cf. *fleischeri* and *B. albifolia*, but both species are easily differentiated even under a dissecting microscope.

Other observed morphotypes tentatively referred to as *B.* cf. *fleischeri* are briefly discussed below.

Specimen Cam-81–11–11.

Comment 3. Quite similar to Cam-89–10–11, but peculiar by more deeply (more distinctly) lobed leaves. In the latter respect seems to be morphologically similar to DAK1 valezh1 kushn6, but small-celled rim is prominent in all the parts of leaf margin. The plants in the specimen besides are characterized by larger leaves those are more or less distinctly falcate.

Specimen Cam-83–16–11.

Comment 4. Brownish to brown colored plants highly similar to plants in Cam-89–10–11, except of distinctly lobed leaves (versus only very shortly lobed) in the present specimen.

Specimen Cam-81–1–11.

Comment 5. Plants rigid, brown to yellowish brown, with small cells present in antical leaf margin, but not so in the postical margin. Cuticle in the apical part of the leaf finely verrucose to papillose, leaves distinctly trilobed.

Specimen Cam-84–7–11.

Comment 6. Plants brownish to brown. Small-celled zone present only along antical margin, not in the postical. Leaves distinctly trilobed.

Although we did not see the plants we could send to the ‘true’ *B. fleischeri*, below we provide the general distribution data of the taxon with some reservation for the unclear taxonomic position of our specimens.

**Figure 15:**
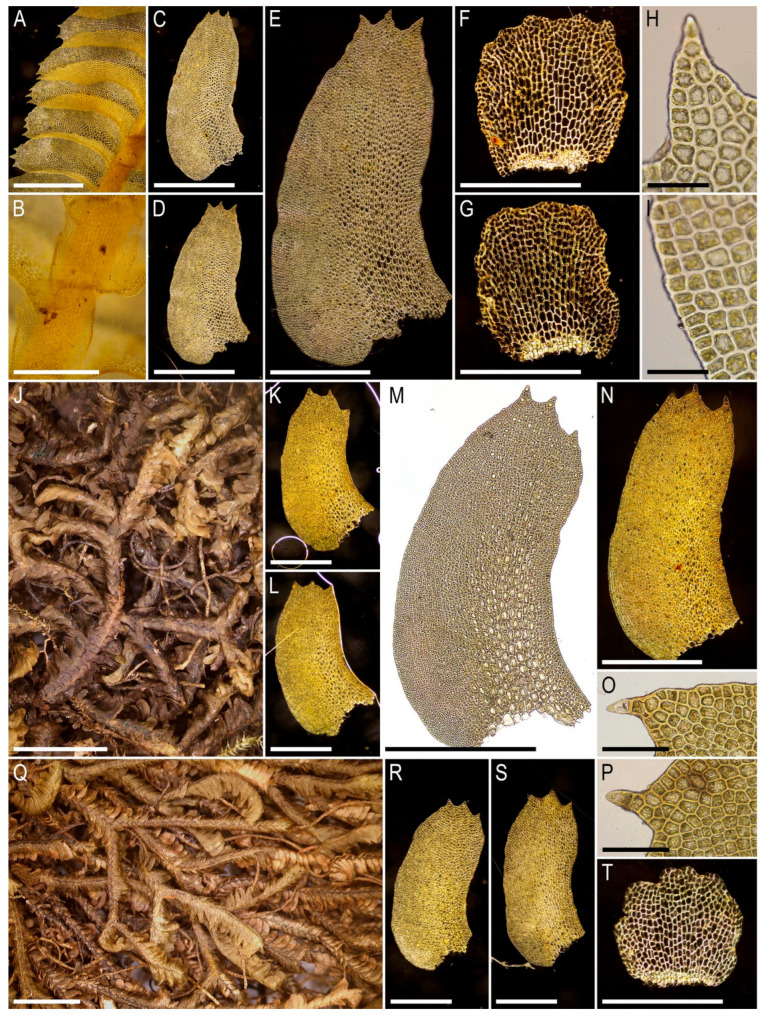
*Bazzania fleischeri* (Steph.) Abeyw.: (**A**) leaves on a shoot, ventral view; (**B**) underleaves on a shoot, ventral view; (**C**–**E**,**K**–**N**,**R**,**S**) leaves; (**F**,**G**,**T**) underleaves; (**H**,**O**,**P**) cells of leaf lobe; (**I**) cells of leaf margin; (**J**,**Q**) parts of mat. (**A**–**G**,**K**,**L**,**N**,**R**–**T**) are photographed with dark field option. Scales: 3 mm for (**J**,**Q**); 1 mm for (**A**,**C**,**D**); 500 µm for (**B**,**E**–**G**,**K**–**N**,**R**–**T**); 100 µm for (**O**,**P**); 50 µm for (**H**,**I**). (**A**–**I**) from Cam-81–11–11, (**J**–**M**) from Cam-83-16-11, (**N**–**P**) from Cam-81-1-11, (**Q**–**T**) from Cam-84-7-11 (VBGI).

Illustrations in the present paper: Figure 15.

Distribution. South Asia (Sri Lanka), East Asia (China: Hainan) [26]. Stephani [45] has reported it for Sino-Himalaya (Assam), but due to Mizutani [13] the latter report is uncertain. Due to the present observation, the species is newly reported from Indochina (Vietnam and Cambodia).

Ecology. In China the species is reported from decaying wood in evergreen forests below 1000 m alt. [26]. The ecology in Cambodia is slightly different: the species is occurring in lower elevations (below 700 m a.s.l.) on tree trunks and once also collected on the stone in dry (wetted in the flood period) riverbed. However, the occurrence in Vietnam is from decaying wood at the elevation slightly above 1000 m a.s.l. All the observed occurrences are from semidry evergreen forest.

Specimens examined: CAMBODIA. Koh Kong Province, Tatai Waterfall (11.58639° N 103.09639° E), 400 m a.s.l., area near the waterfall in evergreen forest, stone near the riverbed, 26 December 2011, V.A. Bakalin, Cam-89–10–11 (VBGI); VIETNAM. Central Highlands, Gia Lai Province, Kbang District (14.48959800° N 108.56691800° E), 1015 m a.s.l., evergreen tropical forest with substantial share *Dacrydium elatum*, large dead trunk, not *Dacrydium elatum* (wood was examined), 7 March 2022, E.V. Kushnevskaya, DAK1 valezh1 kushn6 (VBGI); *ibid.* (14.48968400° N 108.56652400° E), 1015 m a.s.l., evergreen tropical forest with substantial share *Dacrydium elatum*, on dead wood of middle stages of decomposition, mixed with *Bazzania tridens* (Reinw., Blume et Nees) Trevis., *Bazzania* albifolia, 14 March 2022, E.V. Kushnevskaya, DAK9 valezh2 kushn3 (VBGI).

Specimens examined for deviations: CAMBODIA. Koh Kong Province, Phumi Chréav (11.80528° N 103.51083° E), 700 m a.s.l., broadleaved evergreen forest on slope, tree trunk, 22 December 2011, V.A. Bakalin, Cam-81–1–11, Cam-81–11–11 (VBGI); *ibid.* Thma Bang Village area (11.66194° N 103.39861° E), 360 m a.s.l., broadleaved evergreen forest on slope to waterfall, tree trunk, 23 December 2011, V.A. Bakalin, Cam-83–16–11 (VBGI); *ibid.* (11.68750° N 103.42722° E), 400 m a.s.l., broadleaved evergreen forest on slope to waterfall, bark of tree; 23 December 2011, V.A. Bakalin, Cam-84–7–11 (VBGI).

*Bazzania intermedia* (Gottsche et Lindenb.) Trevis., Mem. Reale Ist. Lombardo Sci. (Ser. 3), C. Sci. Mat. 4 (13): 415, 1877

Descriptions: [41,47].

Illustrations: [41] (Figure 20), [47] (Plate XV, Figures 16–25), [13] (Figure VIII).

Description. Plants yellowish brownish, freely branched (*Frullania*-type), ventral scale-like leaved flagella abundant, 15–30 mm long and 2.2–2.8 mm wide. Rhizoids virtually absent even at the distal parts of flagella. Leaves obliquely inserted, slightly overlapping (covering up to 1/3 in the leaf base of the next situated leaf), slightly ridged to plane in upper halves, somewhat falcate, when flattened in the slide 1400–1800 × 600–900 µm, distinctly 3–lobed, in upper half of the leaf with solitary to numerous accessory teeth, cells in the middle of the lower half of the leaf are longer, but leaves are not vittate. Midleaf cells distinctly longer than along the margin, (20–)25–45 × 13–20 µm, thin-walled, with moderate to large in size, slightly concave to convex trigones, cuticle papillose; in dorsal leaf margin 8–18 µm, with unclearly thickened walls and small to moderate in size, concave trigones, cuticle papillose-verrucose, mostly unclearly so. Underleaves 1.5 wider than the stem, appressed in lower parts (where chlorophyllose) and obliquely spreading to loosely recurved above, longer than wide, when flattened in the slide 500–600 × 400–500 µm, marginal rows of discolored cells are with thin walls, middle part (actually the chlorophyllose zone is very variable in the size and shape) with thick cell walls and distinct trigones, loosely connate or adjacent to the lateral leaves in the both sides.

**Figure 16:**
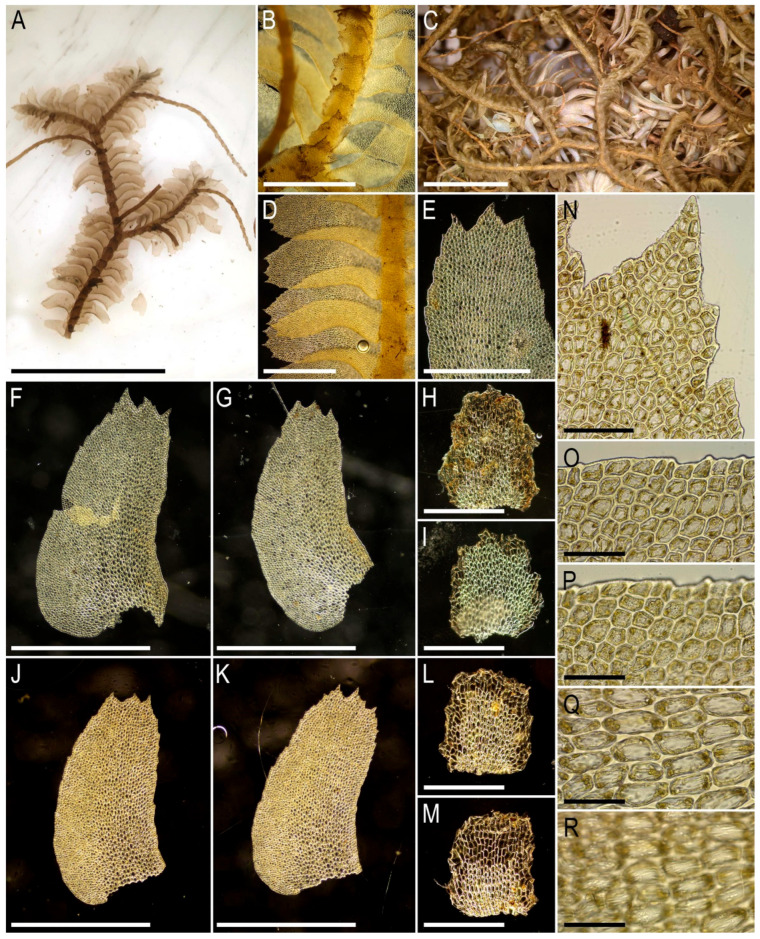
*Bazzania intermedia* (Gottsche et Lindenb.) Trevis.: (**A**) shoot, ventral view; (**B**,**D**) part of shoot, ventral view; (**C**) part of mat; (**E**) upper part of the leaf; (**F**,**G**,**J**,**K**) leaves; (**H**,**I**,**L**,**M**) underleaves; (**N**) cells of leaf lobe; (**O**,**P**) cells in dorsal leaf margin; (**P**) papillose–verrucose cuticle of dorsal leaf margin; (**Q**,**R**) midleaf cells; (**R**) papillose cuticle in midleaf area. (**B**,**D**–**M**) are photographed with dark field option. Scales: 6 mm for (**A**); 5 mm for (**C**); 1 mm for (**B**,**D**,**F**–**M**); 500 µm for (**E**); 50 µm for (**N**–**R**). (**A**,**B**,**E**–**I**,**N**–**R**) from Cam-84-8-11, (**C**,**D**,**J**–**M**) from Cam-84-18-11 (VBGI).

Illustrations in the present paper can be found in Figure 16.

Comment. In many aspects, the species somewhat resembles *B. tridens*, but is easily recognizable due to its only partly hyaline underleaves (lower half chlorophyllose) and commonly numerous accessory teeth in the apical parts. The species is somewhat similar to *B. adnexa*, which, however, differs in its distinctly wider than long underleaves. Some deviations were observed in some plants in our specimens: (1) underleaves may be almost completely hyaline, thus approaching *B. tridens*, although the leaves have numerous accessory teeth; (2) the underleaves are partly hyaline (thus, as it ‘should be’), but leaves have solitary to nearly absent accessory teeth, thus approaching *B. tridens* in this feature; (3) the leaf cuticle varies from papillose to virtually smooth, as observed in *B. tridens*. Furthermore, we now follow Mizutani [13] (p. 88) regarding *Bazzania wallichiana*, which “seems to be closely related to, and may be merely a form of *B. intermedia*”.

Distribution. From South Asia (India: Tamil Nadu State), Southeast Asia (Southern Japan (Ryukyu, Bonin)) through Indochina (Vietnam, Cambodia—new report), Malesia, Melanesia and to Oceania and Australasia [40,41,43,48,49].

Ecology. In the southern Japan Horikawa [43] (p. 196) the species occurs “on the banks, barks and decayed woods”. The ecology of the species in Cambodia partly coincides with the latter. The species occurs in evergreen dense forests on slopes to watercourses, where it grows on the bark of trees and on more or less dry cliffs at lower elevations varying from 360 to 400 m a.s.l.; this is one of the lowest occurrences of the genus in Indochina.

Specimens examined: CAMBODIA. Koh Kong Province, Thma Bang Village area (11.68750° N 103.42722° E), 400 m a.s.l., broadleaved evergreen forest on slope to waterfall, bark of tree; 23 December 2011, V.A. Bakalin, Cam-84–8–11, Cam-84–18–11 (VBGI).

*Bazzania paradoxa* (Sande Lac.) Steph., Bot. Jahrb. Syst. 23 (1/2, 3): 307, 1896

Descriptions: [11,47].

Illustrations: [11] (Table IX), [47] (Plate XVIII, Figures 1–11).

Description. Plants merely rigid, yellowish brownish in the herbarium, regularly branched, ventral scale-like leaved ‘flagella’ numerous, 30–50 mm long and 3–4 mm wide. Leaves obliquely inserted, laterally suberect spreading, slightly convex-ridged, in ventral side with toothed appendage, obliquely ovate, 1800–2200 × 1100–1300 µm. Midleaf cells thin-walled, with large, rarely confluent, nodulose trigones, mostly oblong to shortly so, 25–40 × 15–22 µm, cuticle smooth to indistinctly papillose; cells along dorsal margin 12–25 µm, thin-walled, external wall thickened, trigones large, nodulose, to slightly convex or concave, when adjacent to the external wall, cuticle papillose. Underleaves concave-canaliculate, loosely appressed to narrowly spreading, not or indistinctly connate with leaves in one side, subquadrate, 700–1000 × 700–1000 µm, densely toothed-laciniate, with large and densely toothed basal appendages in the both sides, overlapping 1/3–1/2 of above situated underleaf. Dioicous. Androecia ventral, with 10 and more pairs of bracts, becoming depauperate and appears to be dying at the tips, subisophyllous, but male bracteoles without antheridia. Perianth on abbreviated ventral branch, 3–plicate, ovate-fusiform, 3.8–4.0 mm long and 1.0–1.2 mm wide, mouth laciniate-ciliate, with uniseriate cilia 3–4 cells long; with 3 pairs of bracts, bracts and bracteoles indistinguishable, innermost narrowly ovate, dentate-ciliate-laciniate through the margin, shortly bilobed in the apex, 1.7–2.0 × 1.0–1.2 mm, middle pair of bracts deeply bilobed, ciliate-laciniate through the margin, 1.0–0.7–0.9 mm, outer bracts shortly to deeply bilobed, more or less ovate, 0.5–0.7 × 0.4–0.6 mm. Capsule short emerged from the perianth, with 4 valves, valve length ca. 1 mm. Seta 4 mm long. Elaters mostly sessile on the apical and lateral parts of the inner side of capsule, bispiral, without homogenous ends, 300–500 µm long and (6–)8–10 µm wide. Spores spherical, brown, finely papillose, 23–26 µm in diameter, outer ells of the capsule ca. 23–25 µm wide, the length is difficult to measure since the cells are mostly collapsed with transverse walls difficult to find.

**Figure 17:**
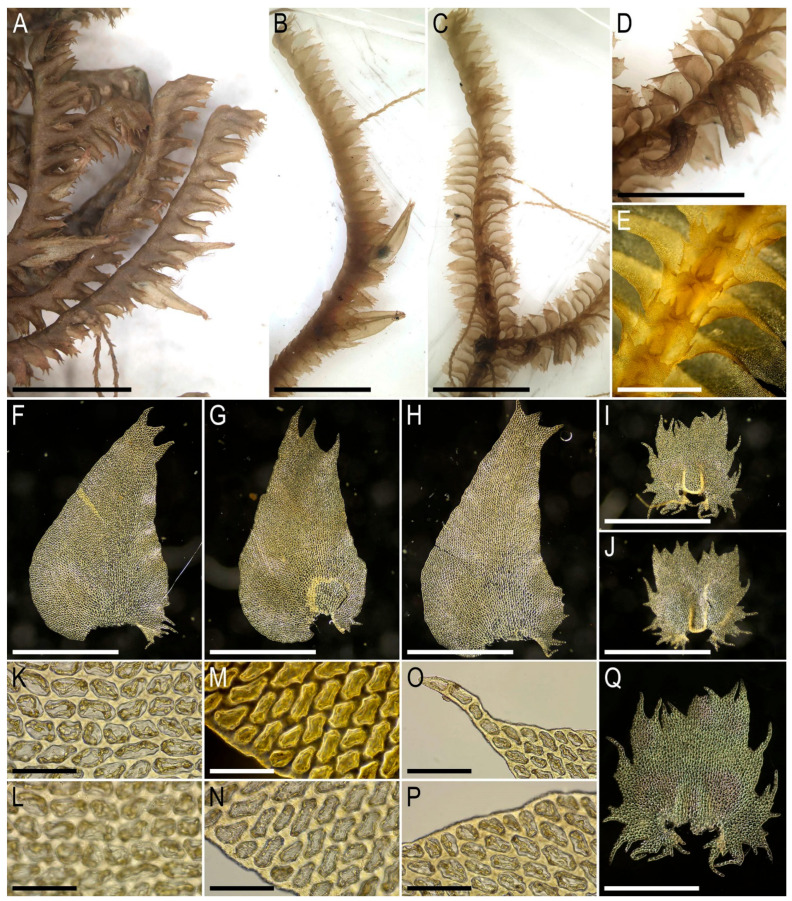
*Bazzania paradoxa* (Sande Lac.) Steph.: (**A**,**B**) shoots with perianths, lateral view; (**C**) shoot with androecia, ventral view; (**D**) part of shoot with androecia, ventral view; (**E**) part of shoot, ventral view; (**F**–**H**) leaves; (**I**,**J**,**Q**) underleaves; (**K**,**L**) midleaf cells; (**L**) smooth to indistinctly papillose cuticle in midleaf area; (**M**,**N**,**P**) cells in leaf margin; (**N**) papillose cuticle in leaf margin area; (**O**) underleaf lacina. (**F**–**J**,**Q**) are photographed with dark field option. Scales: 5 mm for (**A**); 4 mm for (**B**,**C**); 3 mm for (**D**); 1 mm for (**F**–**J**); 500 µm for (**Q**); 50 µm for (**K**–**P**). (**A**,**F**,**H**,**M**,**N**) from Cam-87-39-11, (**B**–**E**,**G**,**I**–**L**,**O**–**Q**) from Cam-87-38-11 (VBGI).

**Figure 18:**
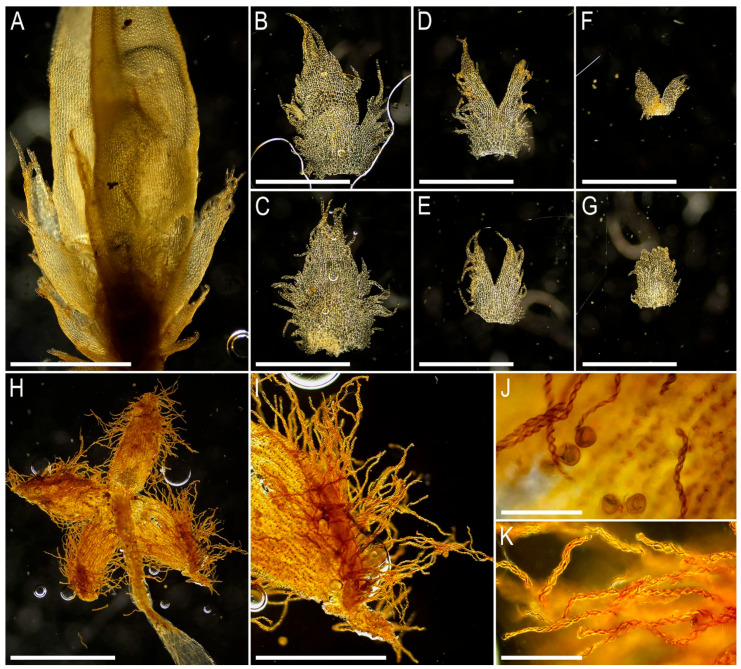
*Bazzania paradoxa* (Sande Lac.) Steph.: (**A**) perianth; (**B**,**C**) inner bracts; (**D**,**E**) middle bracts; (**F**,**G**) outer bracts; (**H**) open capsule; (**I**) capsule lobe with elaters; (**J**) spores and elaters on the inner surface of capsule lobe; (**K**) elaters. All are photographed with dark field option. Scales: 1 mm for (**A**–**H**); 500 µm for (**I**); 100 µm for (**J**,**K**). All from Cam-87-38-11 (VBGI).

Illustrations in the present paper can be found in Figure 17 and Figure 18.

Comment. The taxon may be mistaken with *B. calcarata* (Sande Lac.) Schiffn., from which it is differing in several features:

1. The underleaves overlap the next nearest underleaf for 1/3–1/2 of their lengths, whereas they are distanced in *B. calcarata* (as discussed by Kitagawa [19]);
2. The plant size is 3.0–4.0 mm wide, versus 2.4–3.0 mm;
3. The leaf cell trigones abruptly end in *B. calcarata* (as may be evidenced from Kitagawa’s [19] pictures), but have a more or less smooth gradient to the cell wall in *B. paradoxa*;
4. The appendages are present in the underleaf bases, versus absent or poorly developed in *B. calcarata*, although Kitagawa [19] indicates the presence of appendages;
5. The leaves 1.5 as long as wide, versus ca 2.0 as long as wide in *B. calcarata*;
6. The leaf ventral base has commonly toothed auricles, versus leaf ventral base having a tooth, but not auricles (although Kitagawa [19] highlights small auricles);
7. No teeth in dorsal leaf base, versus dorsal leaf base sometimes with teeth in *B. calcarata*;
8. The leaf cuticle is papillose, sometimes unclearly so, versus smooth in *B. calcarata*.

The name ‘*calcarata*’ seems to mean the occurrence on calcium-rich substrates, while *B. paradoxa* seems to occur on trees. However, *B. calcarata* also occurs on rocky substrates. Tixier [50] (p. 342) provided occurrence as “on rotten wood in evergreen forest” in Pahang, Malaysia.

Despite the numerous listed features, intermediate, hardly distinguishable modifications may occur. For instance, Cam-87–39–11 is characterized by narrower leaves, and more abruptly ended trigones, but possesses a prominently papillose leaf cuticle. The plants in our specimens and the illustrations in the above cited sources are similar to those photographed and published in Khotimperwati et al. (2018) [51] under *B. calcarata*, leading us to suspect that the report in the latter source is misidentification-based.

Distribution. Indochina (Thailand, Cambodia—new report here), Malesia (Malaysia, Indonesia) Melanesia (Fiji), Polynesia (Samoa) [16,23,25,40,50].

Ecology. Siregar et al. [25] provide data on the collection, including climatic measurements at that time. The species occurs in epiphytic habitats at an elevation of 1421 m a.s.l. with humidity near 72% and temperature near 24 °C. Data on ecology in Thailand are more scarce and,, following Kitagawa [16] (p. 265), these are “tree trunk and rock in rain forest”. The species was collected in Cambodia at one site in a wet evergreen forest at the edge of grassland on the bark of tree at an elevation of 380 m a.s.l.

Specimens examined: CAMBODIA. Koh Kong Province, National Mega Forested Area (11.59694° N 103.22556° E), 380 m a.s.l., wet broadleaved evergreen forest at the edge of grassland, bark of tree, 24 December 2011, V.A. Bakalin, Cam-87–38–11, Cam-87–39–11 (VBGI).

*Bazzania recurva* (Mont.) Trevis., Mem. Reale Ist. Lombardo Sci. (Ser. 3), C. Sci. Mat. 4 (13): 414, 1877

Descriptions: [52].

Illustrations: [29] (Figures 4A,B and 6C,D); [53] (Figure 4).

Description. Plants yellowish brownish when dry, in dry condition strongly turned ventrally, but with leaves not clasping, due to this, plants in dry conditions much narrower than in wet conditions and only 1.0–1.3 mm wide, due to imbricate and turned ventrally leaves plants resembling in dry conditions *Acrolejeunea infuscata* (Mitt.) Jian Wang bis & Gradst., but immediately different in numerous ventral ‘flagella’; in moist conditions 40–70 mm long and 3.0–3.5 mm wide, sparsely terminally branched (branching of *Frullania*-type) and with numerous ventral ‘flagella’ with reduced leaved, although some ‘flagella’ has subimbricate and not so reduced leaves. Rhizoids brownish, very sparse, present in terminal parts of ‘flagella’. Leaves obliquely to subtransversely inserted and oriented, suberect spreading, imbricate, convex-ridged, somewhat turned ventrally, in apical part somewhat involute, when flattened in the slide commonly becoming lacerate or plicate, both in ventral and dorsal bases sometimes bear sharply to obtusely dentate appendages, in general outline widely obliquely ovate, unlobed, 1200–1600 × 1700–2000 µm, with prominently denticulate margin with becoming longer protruding to the apex, apex also rarely very shortly emarginate. Midleaf cells subisodiametric, 25–30 × 25–27 µm, thin-walled, with large nearly triangular trigones with visible middle lamina, trigones sometimes confluent in shorter wall, cuticle smooth. Cells along margin 12–20(–25) µm, external wall very thick, tooth situated above middle of the cell lumen, other walls thin to thickened due to trigones confluence, trigones large, concave to convex, sometimes confluent in radial wall, commonly with visible middle lamina, cuticle verrucose. Underleaves canaliculate-concave (if to view from ventral side), imbricate with apical part and sides loosely recurved, although situated very closely to the ventral base of the leaves seems never connate with them, when flattened in the slide commonly becoming lacerate or plicate, virtually without appendages or with very unclear ones, in general outline transversely ellipsoidal, 800–1000 × 1500–1900 µm, prominently denticulate through the margin (more prominently than the leaf sides).

**Figure 19:**
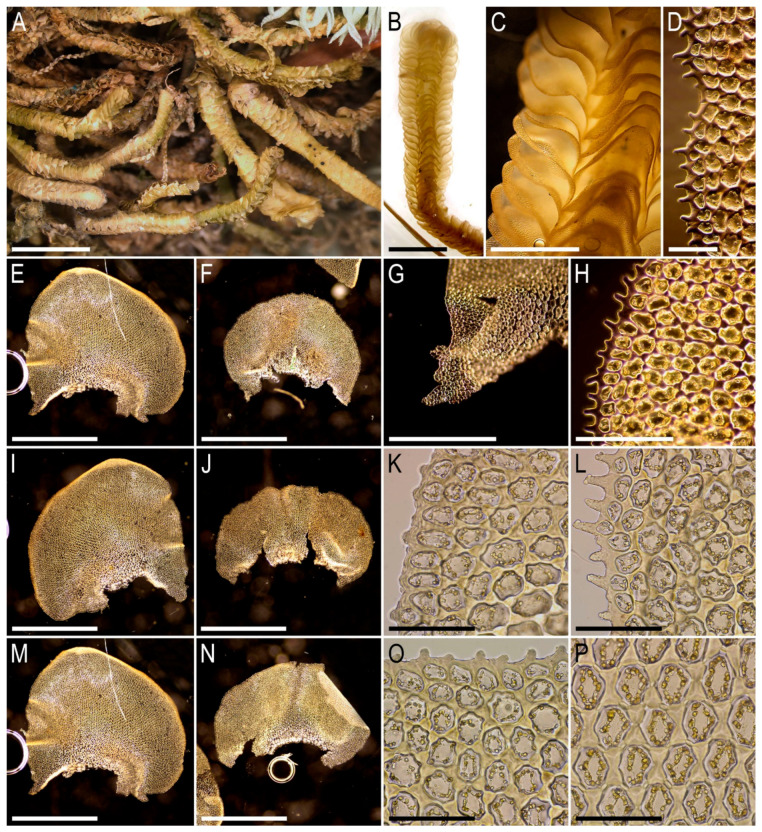
*Bazzania recurva* (Mont.) Trevis.: (**A**) shoots in a part of mat; (**B**) shoot, ventral view; (**C**) part of shoot, ventral view; (**D**) denticulate margin of the leaf; (**E**,**I**,**M**) leaves; (**F**,**J**,**N**) underleaves; (**G**) appendage of the leaf; (**H**) denticulate margin of the underleaf; (**K**,**L**,**O**) cells in leaf margin area, cuticle verrucose; (**P**) midleaf cells. (**C**–**J**,**M**,**N**) are photographed with dark field option. Scales: 3 mm for (**A**,**B**); 1 mm for (**C**,**E**,**F**,**I**,**J**,**M**,**N**); 500 µm for (**G**); 100 µm for (**K**,**L**,**O**,**P**); 50 µm for (**D**,**H**). All from kushn7-epiphytes (VBGI).

Illustrations in the present paper: Figure 19.

Comment. *Bazzania recurva* possesses a peculiar type of denticulation formed entirely by wall thickenings those are not accompanied by cell lumen protrusions. Illustrations of the species are provided by Pócs [29] for Vietnam and Piippo et al. [53] for Singapore. The same authors and Kitagawa [19] mention only selected characteristics of the species; apparently, the species was only described in the original description, so we provide its full description here. Kitagawa [19] (pp. 80–81) noted the close resemblance of *B. recurva* with *B. confertifolia* (Steph.) Herzog (a Malesian-Melanesian species), but the former “the leaves serrulate rather regularly and the underleaves bear no basal auricles”, versus denticulations are scattered and basal auricles well-developed in *B. confertifolia*.

Distribution. Probably widely distributed in SE Asia including Indochina (Vietnam, Thailand) and Malesia (Malaysia) [19,23,29,40,54,55,56].

Ecology. Pócs, 2023 [29], describes the habitat as “sur bois au sol en forêt, 900 m” [on wood on the ground in the forest] (Vietnam, Lâm Đồng, Bao Loc). On the contrary, our specimen is from the fallen branch of *Fokienia hodginsii*, originally situated in a tree crown.

Specimens examined: VIETNAM. Central Highlands, Lâm Đồng Province, Lạc Dương District (12.18141667° N 108.69028333° E), 1550 m a.s.l., evergreen tropical mountain forest with *Fokienia hodginsii* and *Pinus krempfii*, on fresh broken branch of old large *Fokienia hodginsii*, 28 October 2023, E.V. Kushnevskaya, kushn7-epiphytes (VBGI).

*Bazzania vittata* (Gottsche) Trevis., Mem. Reale Ist. Lombardo Sci. (Ser. 3), C. Sci. Mat. 4 (13): 414, 1877

Descriptions: [27,41,42,47].

Illustrations: [27] (Figure 37), [41] (Figure 33), [42] (Figure 13L–P).

Description. Plants brownish-whitish when dry, very fragile, closely adjacent to the substrate, in loose patches, with tiny admixture of *B. intermedia*, 7–15 mm long and 1.0–1.2 mm wide, sparsely branched (*Frullania*-type), ventral scale-like leaved ‘flagella’ sparse. Rhizoids absent in normally leaved shoots but present distal areas of ‘flagella’, where brownish, obliquely to erect spreading originating from the stem just below modified leaves and underleaves. Leaves obliquely inserted and oriented, suberect spreading, slightly convex-ridged (both in dry and wet conditions), imbricate, overlapping ca. 1/5–1/4 of the next leaf in the base, when flattened in the slide obliquely ovate to very slightly falcate, 500–600 × 250–350 µm, with apex shortly, but distinctly trilobed, lobes acute, with vitta formed by 3–4 rows of cells reaching 2/3 of leaf length and sometimes even entering to the upper third of the leaf, without accessory teeth along leaf margin (except of rare aberrations). Cuticle densely verruculose through the leaves and underleaves; vitta cells rectangular, 15–25 × 15–20 µm, with walls slightly thickened, trigones moderate in size, concave; in the midleaf aside vitta cells subisodiametric, 10–13 × 10–13 µm, with thin to somewhat thickened walls, trigones mostly small to moderate in size, concave; cells along margin 10–14 µm, mostly thin-walled, external wall may be slightly thickened, trigones small to moderate, concave. Underleaves tightly appressed to the stem to narrowly spreading in apical third of the underleaf, contiguous to somewhat distant, subquadrate to rectangular, 200–250 × 150–200 µm, hyaline except 1–several rows of cells very near the base, shortly emarginated to bilobed for 1/5 of underleaf length, with rounded lobe apices or shortly and unclearly 3(–4)-lobed, with rounded lobes.

**Figure 20:**
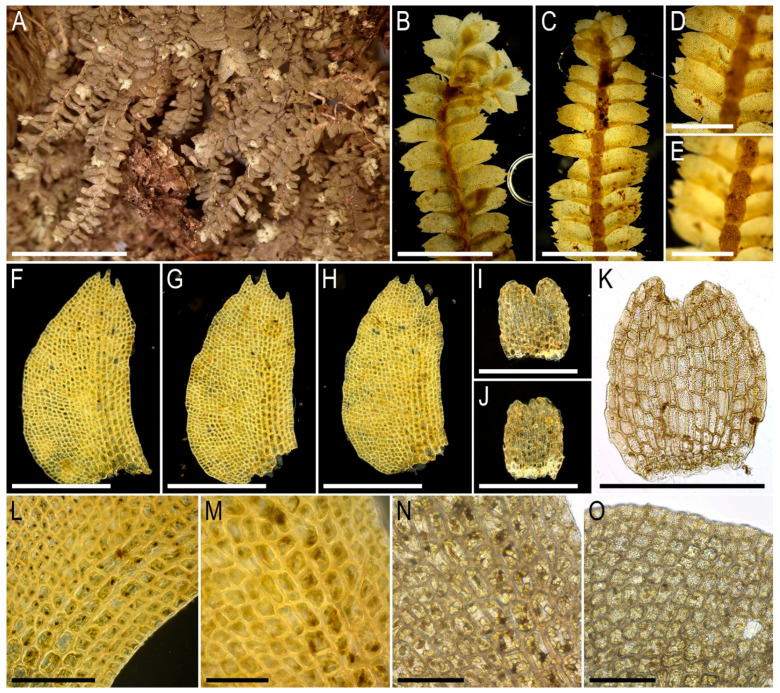
*Bazzania vittata* (Gottsche) Trevis.: (**A**) part of mat; (**B**) shoot, dorsal view; (**C**) shoot, ventral view; (**D**) part of shoot, dorsal view; (**E**) part of shoot, ventral view; (**F**–**H**) leaves; (**I**–**K**) underleaves; (**L**–**N**) vitta cells and aside vitta cells; (**O**) verruculose cuticle. (**B**–**J**,**L**,**M**) are photographed with dark field option. Scales: 3 mm for (**A**); 500 µm for (**B**,**C**); 300 µm for (**F**–**J**); 200 µm for (**K**); 100 µm for (**L**); 50 µm for (**M**–**O**). All from Cam-79–32–11 (VBGI).

Illustrations in the present paper can be found in Figure 20.

Comment. Among the regional species, *B. vittata* is characterized by its (1) densely verruculose cuticle through the leaf and underleaf surfaces, (2) almost completely hyaline underleaves, and (3) distinctly vittate leaves. The lobation of the leaves is the subject of great variation. While modifications with only short to almost unlobed leaves are common, our plants display modification with more distinct than commonly trilobed leaves. Some resemblance may be seen with other small-sized species in the flora—*Bazzania mayebarae* S. Hatt. (to which *B. debilis* N. Kitag. may be identical). *Bazzania mayebarae* is, however, different from *B. vittata* in that the (1) plants are not so densely adjacent to the substrate, (2) have bilobed leaves, (3) an absence of vitta, and (4) a verrucose to papillose, but not verruculose, leaf cuticle. Zhou et al. [27] also stressed some difficulties in differentiating *B. vittata* from *B. tridens* due to the occasional presence of a vitta-like cell group in the central part of the leaves in the latter. However, *B. vittata* is much smaller, has a whitish color and possesses a pronouncedly verruculose leaf cuticle.

Distribution. East Asia (China: Hainan), Indochina (Thailand, Cambodia—the present report), Malesia (Indonesia, Malaysia) to Melanesia (New Guinea), Oceania (Tahiti, New Caledonia) and Australasia [7,27,40,41,42]. The species is hitherto new to Cambodia.

Ecology. Mejer [57] (p. 372) describes the occurrence in Java Island as “in primary forests, … 1200–2700 m”. The bark of trees, fern trees, and rotten wood in 700–2000 m a.s.l. are known as the habitat in the Philippines [7]. Essentially the same as above is the habitat in New Caledonia, where the species occurs on decaying wood, tree fern stripes, and the bark of trees at ca. 500–1400 m a.s.l. [42]. In East Indochina, this seems to be quite a rare species, collected just once on a tree base in an evergreen tropical forest on a slope at an elevation of ca. 900 m a.s.l.

Specimens examined: CAMBODIA. Mondulkiri Province, Phnom Nam Lyr Wildlife Sanctuary (12.54444° N 107.50861° E), 900 m a.s.l., broadleaved evergreen forest on slope, tree base, 19 December 2011, V.A. Bakalin, Cam-79–32–11 (VBGI).

*Lepidozia holorhiza* (Reinw., Blume et Nees) Nees, Syn. Hepat. 2: 210, 1845

Illustrations: [14] (Figure V1–10).

Description. Plants in loose patches, yellowish greenish in the herbarium, pale green when fresh, freely pinnately branched (branching *Frullania*-type), ca. 70–80% of branches becoming at the ends into flagelliform shoots with reduced leaves and in that area branching again (but branches there is only ventral), ventral ‘flagella’ with reduced leaves sparse, but occurs regularly, normally leaved shoots 20–40(–50) mm long and 0.7–0.8(–0.9) mm wide. Leaves imbricate or nearly so, subtransversely inserted and oriented, slightly convex, densely ciliate-laciniate along margin, lacinae become longer to the apex, near apex with 2–3 prominent large lacinae (=lobes), when flattened in the slide always lacerate with dorsal part distinctly larger than ventral part, 500–600 × 600–800 µm. Midleaf cells rectangular, 15–20 × 10–15 µm, walls thin to somewhat thickened, trigones small to moderate in size, concave, cuticle smooth. Uniseriate ends of large lacinae with cells becoming longer to the lacinae apex, 5–7 cells long, the basal cells of uniseriate end start from 20 µm then reaching 50 µm to the apex, external wall thickened, the transverse walls projecting as commonly occurs on *Blepharostoma neglectum* Vilnet & Bakalin, cuticle distinctly papillose in lacinae and cilia. Underleaves erect to suberect spreading, convex to somewhat folded, merely similar to leaves also with 2 prominent lacinae near the apex, but with the sides merely equal in size, always lacerate when flattened in the slides, 450–500 × 500–600 µm.

**Figure 21:**
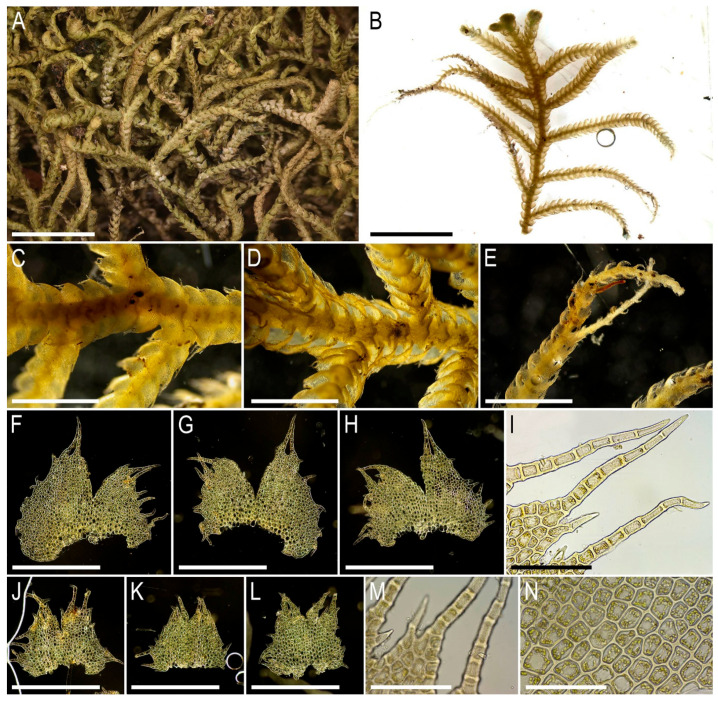
*Lepidozia holorhiza* (Reinw., Blume et Nees) Nees: (**A**) part of mat; (**B**) shoot, dorsal view; (**C**) part of shoot, dorsal view; (**D**) part of shoot, ventral view; (**E**) flagelliform area at the end of the branch; (**F**–**H**) leaves; (**I**) ciliate–laciniate margin of leaf lobe; (**J**–**L**) underleaves; (**M**) papillose cuticle in leaf lobe, lacinae and cilia; (**N**) midleaf cells. (**F**–**H**,**J**–**L**) are photographed with dark field option. Scales: 4 mm for (**A**,**B**); 1 mm for (**C**–**E**); 500 µm for (**F**–**H**,**J**–**L**); 100 µm for (**I**,**M**,**N**). All from V-37-46-22 (VBGI).

Illustrations in the present paper can be found in Figure 21.

Comment. *Lepidozia holorhiza* is morphologically similar to *L. cladorhiza* (Reinw., Blume et Nees) Nees and may be distinguished by the following: (1) leaves that are obliquely trapezoidal, distinctly and long ciliate–laciniate in *L. holorhiza*, versus leaves that are ovate with a rather narrowed apex, without distinct lobation in *L. cladorhiza* and (2) plants narrower than 0.9 mm wide in *L. holorhiza* versus plants wider than 1.0 mm in *L. cladorhiza*.

There are some discrepancies with Mizutani [14]’s description in terms of the length of cilia cells. Following the latter author, *L. holorhiza* cilia cells reach 30 µm long, whereas they reach 90 µm in *L. cladorhiza*. The cilia cells reach 50 µm long in our specimen plants. Another morphologically similar species, *Lepidozia borneensis* Steph. (morphological description and illustrations provided by Mizutani [14], and micrographs by Pócs et al. [30]), differs from *L. holorhiza* in having larger plants (wider than 1.1 mm, versus less than 0.9 mm in *L. holorhiza*) and the presence of short lateral teeth at the base of the underleaf lobes (versus absent in *L. borneensis*).

Distribution. Indochina (Vietnam—the present report), Malesia (Malaysia, Indonesia) [40,58]. The species was reported by Miller et al. [59] for Hawaii in Polynesia, without exact location, and listed in Staples and Imada [60] as dubious records.

Ecology. We did not find data on the ecology of the species in any localities across the species area. The habitat in Vietnam is a mountainous, evergreen *Rhododendron*-tree-dominated forest, at an elevation of 1613 m a.s.l., where the specimen was collected on moist humus on slope in part shade.

Specimens examined: VIETNAM. North Central Coast, Nghệ An Province, Tương Dương District, Pù Mát National Park (19.02464° N 104.58004° E), 1613 m a.s.l., area near the peak of Pù Mát Mt. covered by dense *Rhododendron* forest, moist humus on slope in part shade, 25 May 2022, V.A. Bakalin & M.H. Nguyễn, V-37–46–22 (VBGI, HN).

*Lepidozia miqueliana* Sande Lac., Ann. Mus. Bot. Lugduno-Batavi 1: 301, 1864

Description: [12].

Illustrations: [14] (p. Figure VII1–8); [12] (Plate VII: 1–4).

Description. Plants in loose patches, pure or intermixed with *Bazzania tridens*, *B. vietnamica* Pócs, due to numerous cilia looking at the first glance in the field as *Trichocolea* Dumort. or *Neotrichocolea* S. Hatt. by naked eye, pale green to yellowish greenish when fresh, yellowish-brownish in the herbarium, freely bipinnately branched, 30–50% of the branches of the second order become depauperate with reduced leaves (like flagella) and then dyeing, ventral flagella not seen; main stem with leaves 20–30(–50) mm long and 0.8–1.0 mm wide. Very sparse rhizoid production seen only at the terminal parts of the ‘flagelliform’ branches, where separate, obliquely to erect spreading, nearly brownish to colorless. Leaves subtransversely inserted and oriented, suberect spreading (in the lower half of leaves), strongly convex, covering about 1//3–2/3 of the above situated leaf, when flattened in the slide nearly transversely ellipsoidal in outline, 600–700 × 700–1200 µm, basically unequally 4–lobed (dorsal slightly larger), each lobe commonly subdivided into two sublobes, leaf margin densely ciliate, undivided part (discus) ca. 1/4 of leaf length. Cells I the middle of the discus 25–53 × 25–35 µm, with thin to somewhat thickened walls, trigones small to moderate in size, concave, cuticle smooth. Cells in the ‘sublobes’ 25–30(–40) × 20–25 µm, with thickened walls and moderate to large in size slightly concave to convex trigones with visible middle lamina, cuticle smooth to scarcely verruculose. Cilia 3–5 cells long, with distinctly protruding transverse walls, from 25 µm long in the basal cells to 70 µm long in terminal cells, walls thickened throughout, cuticle smooth. Underleaves suberect spreading (if to look from ventral side), contiguous, although with ciliate apical part covering lower half of the above situated underleaf, 400–500 × 600–750 µm, similar to leaves, but with nearly equal lobes in size, undivided part (discus) ca. 1/3 of the underleaf length.

**Figure 22:**
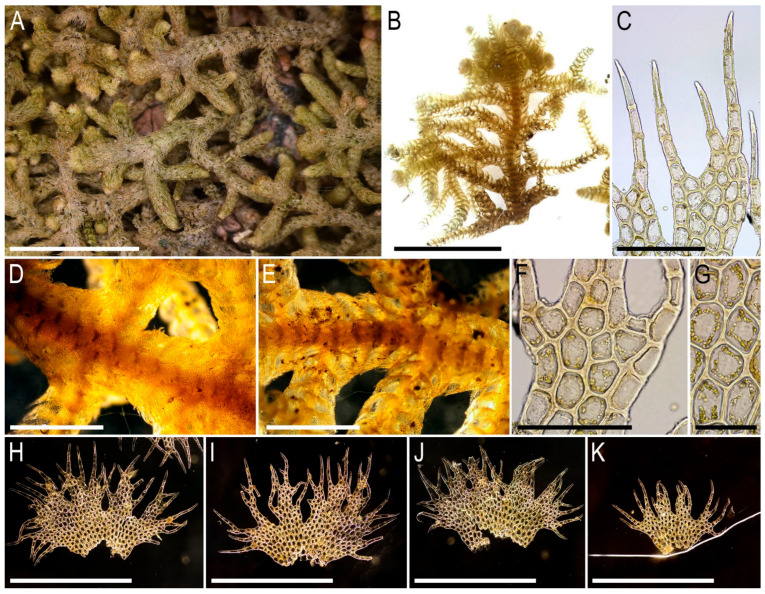
*Lepidozia miqueliana* Sande Lac.: (**A**) part of mat; (**B**) shoot, dorsal view; (**C**) leaf lobes; (**D**) part of shoot, dorsal view; (**E**) part of shoot, ventral view; (**F**) cells of leaf lobe base; (**G**) midleaf cells; (**H**–**J**) leaves; (**K**) underleaf. (**D**,**E**,**H**–**J**) are photographed with dark field option. Scales: 4 mm for (**A**,**B**); 1 mm for (**D**,**E**,**H**–**K**); 100 µm for (**C**,**F**); 50 µm for (**G**). All from V-36–36–22 (VBGI).

Illustrations in the present paper can be found in Figure 22.

Comment. This is a fairly easily recognizable species, resembling *Trichocolea* in the field, either without a magnifying glass or with a weak magnifying glass, due to the large number of cilia along the leaf margin. Mizutani [14] describes and illustrates *Lepidozia lacerifolia* Steph. and points out its close morphological similarity to *L. miqueliana*. However, the latter is characterized by shorter cilia, only 3–5 cells long, and shorter leaf disk cells (22–35 in [14] but 25–53 µm in our specimen). In contrast, *L. lacerifolia* has cilia 5–8 cells long, and the length of leaf disk cells is 50–90 µm.

Distribution. Indochina (Vietnam—the present study); Malesia (Malaysia, Indonesia) [14,40,58].

Ecology. We did not find data on the ecology of the species in any localities of the species area. The habitat in Vietnam is a mountainous, evergreen tropical forest, at an elevation of 1552 m a.s.l., where the specimen was collected on moist humus on slope in part shade. The collection was made a short distance from the collection of another rare taxon treated above (*Lepidozia holorhiza*).

Specimens examined: VIETNAM. North Central Coast, Nghệ An Province, Tương Dương District, Pù Mát National Park (19.02312° N 104.57874° E), 1552 m a.s.l., N-facing slope of Pù Mát Mt. covered by dense tropical forest, moist humus in part shade, 25 May 2022, V.A. Bakalin & M.H. Nguyễn, V-36–36–22 (VBGI, HN).

*Neolepidozia papulosa* (Steph.) Fulford et J. Taylor and *Neolepidozia wallichiana* (Gottsche) Fulford et J. Taylor in East Indochina.

**Figure 23:**
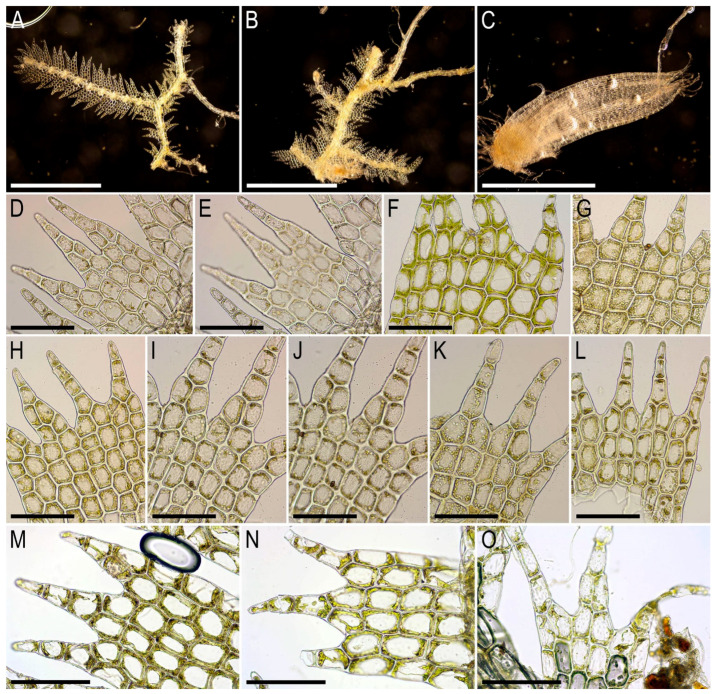
*Neolepidozia papulosa* (Steph.) Fulford et J. Taylor: (**A**,**B**) shoots, dorsal view; (**C**) perianth; (**D**,**E**) leaves attached to the stem; (**F**–**L**) leaves detached of the stem; (**E**,**I**) papillose cuticle on leaf surface. (**A**–**C**) are photographed with dark field option. *Neolepidozia wallichiana* (Gottsche) Fulford et J. Taylor: (**M**–**O**) leaves attached to the stem; (**O**) papillose cuticle on leaf surface. Scales: 1 mm for (**A**–**C**); 100 µm for (**D**–**O**). (**A**–**E**) from DAK1 Valezh1 kushn9, (**F**) from Fok23#2soil kushn14, (**G**) from Cam-81-111-11, (**H**) from Cam-81-109-11, (**I**,**J**) from Cam-87-17-11, (**K**) from Cam-88-8-11, (**L**) from Cam-88-9-11, (**M**) from DAK1 Valezh2 kushn10, (**N**) from DAK1 Derevo1komel kushn12, (**O**) from DAK1 melky debris kushn13 (VBGI).

Illustrations in the present paper can be found in Figure 23.

Comment. Kitagawa [17] first drew attention to *Neopepidozia papulosa* (*Lepidozia papulosa* Steph. in [17]), noting that the frequency of this species is greatly underestimated due to its frequent misidentification as *N. wallichiana*. Describing the distinguishing features, Kitagawa [17] (p. 267) noted the following: “*L. papulosa* and *L. wallichiana* are immediately distinguished from each other in the characteristics of the leaf cells; in *L. papulosa* they are isodiametric or slightly elongated, small (30–40 × 25–30 µm in the middle of discs) and relatively thick-walled, while in *L. wallichiana* they are strongly elongated, much larger (40–60 × 25–35 µm) and thin-walled”. However, even in his description of the delimiting characters, the lower limit of cell length for *Neolepidozia papulosa* (30 µm) is equal to the upper limit of width; that is, the cells cannot be purely isodiametric, at least in most of the material. The second thing that attracts attention is the wide overlap of leaf cell width ranges: 25–30 µm in *N. papulosa* versus 25–35 µm in *N. wallichiana*. Thus, the remaining characteristics are relative cell length (in Kitagawa’s description, the ranges do not overlap) and cell wall thickness (thick versus thin). Observing specimens from central Vietnam, we noted some significant variations in cell size and wall thickness. A description of these variants is provided below and also shown in Figure 23. Thus, the distinction between the two species does not appear as simple as might follow from interpretations of Kitagawa’s [17] explanations. When deciding on species assignment, we essentially had to rely on the cell length-to-width ratio, but even there, significant overlap in variation ranges was noted. The question of the two species’ distinctness can be resolved through molecular genetic analysis of a significant amount of material from various localities of both species, which was not undertaken in this study.

*N. papulosa* (Specimen DAK1 Valezh1 kushn9)

Characterized by midleaf cells 35–45 × 25–35 µm, with somewhat thickened walls, cuticle papillose.

*N. wallichiana* (Specimen DAK1 Valezh2 kushn10)

Midleaf cells 37–50 × 15–35 µm, thin-walled, cuticle smooth.

*N wallichiana* (Specimen DAK1 Derevo1komel kushn12)

Midleaf cells 48–50 × 30–35 µm, thin-walled, cuticle smooth.

*N. wallichiana* (Specimen DAK1 melky debris kushn13)

Midleaf cells 38–45 × 20–28 µm, thin-walled, cuticle papillose

*N. papulosa* (Specimen Cam-81–111–11)

Midleaf cells 30–35 × 30–32 µm, walls slightly thickened, cuticle smooth.

*N. papulosa* (Specimen Cam-88–9–11)

Midleaf cells 40–46 × 20–25 µm, thin-walled, cuticle smooth.

*N. papulosa* (Specimen Cam-88–8–11)

Midleaf cells 40–50 × 25–40 µm, walls thin to thickened, cuticle smooth.

*N. papulosa* (Specimen Cam-85–18–11)

Midleaf cells 25–40 × 25–35 µm, thin-walled, cuticle smooth.

*N. papulosa* (Specimen Cam-81–109–11)

Midleaf cells 25–40 × 25–35 µm, walls thickened, cuticle smooth.

*N. papulosa* (Specimen Cam-87–17–11)

Midleaf cells 35–45 × 30–40 µm, walls thin to thickened, trigones somewhat large, cuticle papillose.

*N. papulosa* (mod. *leptoderma*?) (Specimen Cam-81–110–11)

Midleaf cells 40–60 × 30–45 µm, thin-walled, cuticle smooth.

The ecology of both taxa seems to be very similar when this species is really abundant in the forest community; it may grow on many substrates including soil, woody litter, the bark of the base of trees and fallen logs. On decaying logs, it may be the dominant species. In such cases, the variability of the features increases. Every place both taxa were collected in tropical evergreen forests at the elevations stretched from 200 to slightly above 1000 m a.s.l.

Specimens examined for *Neolepidozia papulosa*: VIETNAM. Central Highlands, Gia Lai Province, Kbang District (14.48938500° N 108.56658100° E), 1026 m a.s.l., evergreen tropical forest with substantial share *Dacrydium elatum*, on large dead fallen trunk, not *Dacrydium elatum* (wood was examined), 7 March 2022, E.V. Kushnevskaya, DAK1 Valezh1 kushn9 (VBGI); North Central Coast, Hà Tĩnh Province, Vũ Quang District, Hương Quang Commune, northern part of Annamite Range (Trường Sơn Range), Vũ Quang National Park (18.24689° N 105.34067° E), 1204 m a.s.l., medium montane evergreen forest on NE-facing gentle slope, rotten part of decaying wood, moist, in part shade, 21 April 2025, K.G. Klimova, V.A. Bakalin and M.H. Nguyễn, Viet-19-8-25 (VBGI, NH); CAMBODIA. Koh Kong Province, Phumi Chréav (11.80528° N 103.51083° E), 700 m a.s.l., broadleaved evergreen forest on slope, fine soil on slope, 22 December 2011, V.A. Bakalin, Cam-81–109–11, Cam-81–110–11, Cam-81–111–11 (VBGI); *ibid*. (11.81556° N 103.47611° E), 700 m a.s.l., broadleaved evergreen forest on slope, fern tree trunk, 25 December 2011, V.A. Bakalin, Cam-88–8–11, Cam-88–9–11 (VBGI); *ibid*. National Mega Forest Area (11.62583° N 103.26861° E), 200 m a.s.l., broadleaved evergreen forest on slope, decaying wood, 24 December 2011, V.A. Bakalin, Cam-85–18–11 (VBGI); *ibid*. (11.59694° N 103.22556° E), 380 m a.s.l., wet broadleaved evergreen forest at the edge of grassland, forest floor, 24 December 2011, V.A. Bakalin, Cam-87–17–11 (VBGI).

Specimens examined for *Neolepidozia wallichiana*: VIETNAM. Central Highlands, Gia Lai Province, Kbang District (14.48938500° N 108.56658100° E), 1026 m a.s.l., evergreen tropical forest with substantial share *Dacrydium elatum*, on deadwood late stages of decomposition, 7 March 2022, E.V. Kushnevskaya, DAK1 Valezh2 kushn10 (VBGI); *ibid.* on bark in the base of large alive tree, 7 March 2022, E.V. Kushnevskaya, DAK1 Derevo1komel kushn12 (VBGI); *ibid.* on small wood debris, on fragments of wood and bark, 7 March 2022, E.V. Kushnevskaya, DAK1 melky debris kushn13 (VBGI); Central Highlands, Lâm Đồng Province, Lạc Dương District (12.18226000° N 108.68964600° E), 1520 m a.s.l., evergreen tropical mountain forest with *Fokienia hodginsii* and *Pinus krempfii*, on open soil, 28 October 2023, E.V. Kushnevskaya, Fok23#2soil kushn14 (VBGI).

*Tricholepidozia semperiana* (Steph.) E.D.Cooper, Phytotaxa 97 (2): 60, 2013

≡ *Telaranea semperiana* (Steph.) Del Ros., Philipp. J. Sci. 100: 238. 1973 (1971).

≡ *Lepidozia semperiana* Steph., Spec. Hep. 3: 612. 1909.

Descriptions: [24,39].

Illustrations: [61] (Figures 39–42); [24] (Figures 11–16 and 22–25).

**Figure 24:**
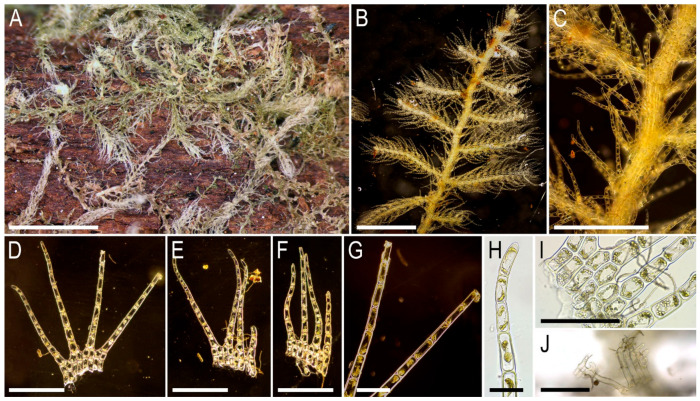
*Tricholepidozia semperiana* (Steph.) E.D. Cooper: (**A**) part of mat; (**B**) shoot, dorsal view; (**C**) part of shoot, dorsal view; (**D**) leaf; (**E**,**F**) underleaves; (**G**,**H**) cells of leaf lobes; (**I**) cells of leaf base; (**J**) rhizoids. (**B**–**G**) are photographed with dark field option. Scales: 1 mm for (**A**,**B**); 500 µm for (**C**); 200 µm for (**D**–**F**); 100 µm for (**I**,**J**); 50 µm for (**G**); 30 µm for (**H**). All from DAK9 valezh2 kushn15 (VBGI).

Illustrations in the present paper can be found in Figure 24.

Comment. A description and detailed illustrations were provided by Shi and Zhu [24], who also described the general distribution of the species (p. 131 in l.c.) and provided a key to the species in Southeast Asia. *Tricholepidozia semperiana* differs from another representative of the genus known in Vietnam (*T. neesii* (Lindenb.) E.D. Cooper) in 4-lobed leaves (versus 6-lobed). The species was collected from several localities on decaying wood, a habitat very characteristic of the taxon.

Distribution. South Asia (Sri Lanka), East Asia (Hainan), Southeast Asia (Vietnam—the present study), Malesia (Philippines, Malaysia) [24,39,40].

Ecology. The species was reported in Hainan, where it was found on a rotten log at an elevation of 950 m a.s.l. [24]. Our specimen was collected in a similar habitat (decaying wood) in an evergreen tropical forest with a substantial share of *Dacrydium elatum*.

Specimens examined: VIETNAM. Central Highlands, Gia Lai Province, Kbang District (14.48968400° N 108.56652400° E), 1015 m a.s.l., evergreen tropical forest with substantial share *Dacrydium elatum*, on dead wood, middle stages of decomposition, 14 March 2022, E.V. Kushnevskaya, DAK9 valezh2 kushn15 (VBGI); North Central Coast, Quang Tri Province, Bố Trạch District, Tân Trạch Commune, northern part of Annamite Range (Trường Sơn Range), buffer zone of Phong Nha–Kẻ Bàng National Park, surroundings of Ba Reng Mt. (17.48958° N 106.37836° E), 818 m a.s.l., small stream with sluggishly flowing water in shady narrow valley surrounded by low tropical montane evergreen forest with major broadleaf species and tree ferns and giant ferns in understory, partly shaded moist fallen decaying decorticated tree trunk, 27 April 2025, V.A. Bakalin, K.G. Klimova & M.H. Nguyễn, V-26-3-25 (VBGI, NH); *ibid*. Hà Tĩnh Province, Vũ Quang District, Hương Quang Commune, northern part of Annamite Range (Trường Sơn Range), Vũ Quang National Park (18.24845° N 105.34189° E), 1117 m a.s.l., medium montane evergreen forest on a small hill, partly shaded mesic fallen decaying decorticated tree trunk, 21 April 2025, V.A. Bakalin, K.G. Klimova & M.H. Nguyễn, V-9-7-25 (VBGI, NH).

## 3. Discussion

Based on the data from the literature provided in the introduction and the additions from the present study, the diversity of Lepidoziaceae in East Indochina (Cambodia, Laos, and Vietnam) consists of the following:

*Acromastigum*—five species. Although we collected several specimens of this genus, we did not treat it here because the distinctions between *Acromastigum herzogii* Grolle and some related taxa require further study.

*Arachniopsis*—one species.

*Bazzania*—36 species, with most known from the northern part of Vietnam and Central Highlands, as well as Lâm Đồng Province of Central Vietnam (the Central Highlands administrative region).

*Kurzia*—five species.

*Lepidozia*—9 species.

*Neolepidozia*—two species.

*Tricholepidozia*—two species.

Thailand is located closest to East Indochina (it borders the studied region to the west). The following number of species are known for it [55]: *Acromastigum* (1 species), *Bazzania* (34), *Kurzia* (4), *Lepidozia* (5), *Neolepidozia* (1), *Tricholepidozia* (1). We assume that the Lepidoziaceae of Thailand may be as understudied as those of Eastern Indochina.

Territorially similar Malaysia [40] contains the following numbers of taxa in the same genera: *Acromastigum* (16 species), *Bazzania* (61), *Kurzia* (6), *Lepidozia* (18), *Neolepidozia* (5), *Telaranea* (including *Arachniopsis major* and *Telaranea papulosa* (Steph.) J.J. Engel et G.L. Merr., which are homotypic synonyms of *Neolepidozia papulosa* but listed in [40] twice under these two names)—3, and *Tricholepidozia*—5 species. If these numbers are compared, it is obvious that further studies will add several new records for Indochinese flora. However, the number of taxa known in East Indochina will hardly reach similar parameters to those in Malaysia because of the considerably wetter climate in the latter [62,63].

One taxon was described as new-for-science (*Bazzania appendiculata* subsp. *cambodiana*), but its status is questionable because a genetic comparison was not conducted. Overall, Lepidoziaceae, as we previously reported [4], may be promising for the discovery of new taxa if molecular genetic methods are used to differentiate species. However, it should be noted that even fresh, visually well-preserved material is usually difficult to analyze, beginning with the extraction of total DNA and the amplification of certain loci. Nevertheless, further work in this area is necessary, and integrative morpho-molecular studies will reveal even more taxa that are new to science.

The results of this study, together with previously published materials, reveal a certain degree of differentiation along the latitudinal gradient of the composition of *Bazzania* in Vietnam. In particular, a number of species not found in northern Vietnam are found in central Vietnam, and vice versa. Currently, 12 species are known in central Vietnam but have not yet been found further north (*Bazzania adnexa*, *B. albifolia*, *B. commutata*, *B. erosa*, *B*. aff. *friabilis* N. Kitag. et Kodama, *B. indica*, *B. loricata* (Reinw., Blume et Nees) Trevis., *B. pectinata* (Lindenb. et Gottsche) Schiffn., *B. recurva*, *B. tranninhiana* Pócs & Gyarmati, *B. spiralis* (Reinw., Blume et Nees) Meijer, *B. vittata*). Moreover, five species have not yet been found in the south (*B. appendiculata*, *B. assamica* (Stephani) S. Hatt., *B. himalayana* (Mitt.) Schiffn., *B. magna* Horik., *B. oshimensis* (Steph.) Horik.). The probability of species known in Central Vietnam occurring in the north can be questioned. However, species currently known from northern Vietnam will likely be found further south in future research within the country.

Although this paper presents a number of species new to the study area, the process of understanding the Lepidoziaceae taxonomic diversity in East Indochina is far from complete. Traditional anatomical and morphological methods may reveal several more taxa that are new to the region, not to mention the fact that the clarification of distribution patterns for taxa already known here will subsequently be significantly refined. An obvious shortcoming of our work is the lack of molecular genetic analysis. When selecting specimens for description, we mostly deliberately discarded morphologically transitional variants, for which it is impossible to draw a definitive conclusion regarding whether they represent a deviant morphotype or a new taxon of some rank. Molecular genetic analysis of Lepidoziaceae is quite difficult due to the suggested DNA methylation; however, we are working on this issue and hope to soon present a more or less comprehensive phylogeny for representatives of the family based on Indochinese materials.

## 4. Materials and Methods

### 4.1. Study Area

The study involves materials collected in East Indochina, as estimated in the present account, covering Laos, Cambodia, and Vietnam. This territory is exceptionally rich in vascular plants [64], and is globally important phytogeographically as a link between the Sino-Himalayan and Malesian floras [4,10,65,66,67]. In the north of the study area are the generally highest elevations of Indochina (the highest point is Phan Xi Păng Mt., 3143 m above sea level); this is a geomorphological continuation of the Hengduan Mountains. South of it, the Annamite Range, a kind of backbone of East Indochina (https://d2ouvy59p0dg6k.cloudfront.net/downloads/greaterannamiteecoregion.pdf (accessed on 15 January 2026)), extends across the study area. Here, a gradient change in mountain flora occurs, and the influence of the Sino-Himalayan mountain region is gradually lost, while its own floristic complex appears. In the south, this complex connects with the extremely distinctive Lâm Viên (Đà Lạt) Plateau [68].

### 4.2. Collecting and Initial Specimen Treatment

All materials were properly labeled at the time of collection, including geographic coordinates, altitude, community type, and substrate. Since some collectors were not professional hepaticologists, and for organizational reasons, the vast majority of the specimens were dried after collection, and oil body data were generally not considered.

### 4.3. Laboratory Treatment

The study was conducted via anatomical and morphological comparisons of specimens with type specimens and descriptions available in the literature, as appropriately cited in the discussion. The structure of the species discussion consists of a nomenclatural citation, data on illustrations and descriptions available in the literature, a morphological description (for most, but not all, species), comments regarding the morphological features of the specimens, a distinction of the discussed species from morphologically similar taxa, distribution, and ecology, and a list of the specimens examined. In all cases, the taxa discussed are accompanied by photographs taken using cameras associated with an Olympus, Olympus corporation, China, Guangzhou CX43 (upright compound microscope) and an Olympus SZX16 (dissecting microscope). The first microscope is equipped with an ADF STD16 camera ADF Optics, China, Shenzhen, and the second with an ADF PRO20 ADF Optics, China, Shenzhen. Both were connected to the computer operating system using the ADF Image Capture software v.4.1.x supplied with the cameras. All materials used have been incorporated into the VBGI herbarium. In general, the approach to the work is of an anatomical and morphological nature and corresponds to that adopted in other studies on the *Lepidoziaceae* family, reflected in a number of literary sources [2,5,6,7,13,14,16,18,19,23,24,26,27,28,29]. In many cases, the morphology of the taxa permits their clear identification, although some taxa need further consideration using molecular analysis, which we did not perform in the present study.

### 4.4. Precautions Regarding Morphological Descriptions

Before reading the descriptions and comments on the species, the following preliminary remarks should be taken into account:

1. Marginal cell measurements are taken from the middle part of the dorsal (antical) leaf margin, if otherwise not mentioned;
2. Underleaf position and shape are described as they appear from the ventral side view (e.g., concave from the ventral side is convex from the imaginary dorsal side view);
3. The verruculose (finely punctate) cuticle is a special feature that is not similar to the terms verrucose or papillose, and resembles very fine-grained dust on the surface of leaves that cannot be washed off with water. Each ‘dust’ particle is less than 0.2 µm in diameter, which is very different from papillae or verrucae, which are ellipsoidal or rounded formations with a smallest dimension of at least 0.8–1.0 µm;
4. Measurements and shape morphology are provided for moistened plants, unless otherwise mentioned;
5. All measurements of leaves, underleaves, and cells are provided first in terms of length and then width and are taken from the main stem, if otherwise not mentioned;
6. The cells in the leaves of all species of *Bazzania* are larger in the midleaf than at the margins. However, the degree of differentiation varies strongly from species to species. This leads to the description of various variants as the large-celled zones or pseudovittate zones in the leaves. Therefore, definitions for these terms need to be provided. Here, we used the term ‘large-celled zone’ for areas where the cell length exceeded the width by less than 1.5 times on average. The term ‘pseudovittate’ is applied when the length of the cells exceeds the width by more than 1.5 times on average, with at least some cells whose length exceeds their width by 2 times. The term vitta is applied for the cases (in the treated taxa only *B. vittata*) where long cells form well-defined structures and thus clearly differ from surrounding cells. In fact, there is a distinct intergradation between all these types, and the differences look merely quantitative.

## 5. Conclusions

In the course of this exploration, one taxon (*Bazzania appendiculata* subsp. *cambodiana*) is described as new-for-science and one genus (*Arachniopsis*) is reported for the first time in Indochina, while five species are new reports for Cambodia, seven for Vietnam, and one for Laos. Since all new findings were made in mountainous areas, it can be expected that further exploration of previously unexplored and poorly studied mountain ranges will reveal a number of additional species. Given the new reports identified in this study, which were primarily based on the collections of nonprofessional hepaticologists, we can expect new discoveries in all the genera of Lepidoziaceae already known from Indochina. One of the most important tasks for further work should be the involvement of molecular genetic analysis, despite the difficulties encountered when using traditional methods for conducting such analysis. Continued research will bring us closer to understanding the diversity of this family in Indochina as a whole and will allow us to compile a comprehensive revision of this family for East Indochina, where liverwort flora have been the subject of focused research in recent decades.

## Figures and Tables

**Table 1:** Summary of treated Leidoziaceae taxa.

Name	Novelty	Distribution in East Indochina	General Distribution Pattern
*Arachniopsis major* Herzog	New genus for Indochina	Vietnam	South Asia to Oceania
*Bazzania adnexa* (Lehm. et Lindenb.) Trevis.	New locality	Vietnam	Southern East Asia and Indochina to Australasia
*Bazzania albifolia* Horik.	New for Vietnam	Cambodia, Vietnam	East Asia and Indochina to Malesia
*Bazzania appendiculata* (Mitt.) S.Hatt. subsp. *appendiculata*	New for Cambodia, Vietnam	Cambodia, Vietnam	South Asia to Malesia
*Bazzania appendiculata* subsp. *cambodiana* Bakalin, S.S. Choi, Klimova subsp. nov.	New subspecies	Cambodia	Indochina
*Bazzania callida* (Sande Lac. ex Steph.) Abeyw.	New for Laos, Vietnam	Cambodia, Laos, Vietnam	South Asia and Indochina
*Bazzania commutata* (Lindenb. et Gottsche) Schiffn.	New for Vietnam	Vietnam	Indochina to Malesia
*Bazzania erosa* (Reinw., Blume et Nees) Trevis.	New for Cambodia	Cambodia, Vietnam	South Asia, southern East Asia, then Indochina to Oceania
*Bazzania fleischeri* (Steph.) Abeyw.	New for Cambodia, Vietnam	Cambodia, Vietnam	South Asia, southern East Asia and Indochina
*Bazzania intermedia* (Gottsche et Lindenb.) Trevis.	New for Cambodia	Cambodia, Vietnam	South Asia to Australasia
*Bazzania paradoxa* (Sande Lac.) Steph.	New for Cambodia	Cambodia	Indochina to Polynesia
*Bazzania recurva* (Mont.) Trevis.	New locality	Vietnam	Indochina to Malesia
*Bazzania vittata* (Gottsche) Trevis.	New for Cambodia	Cambodia	East Asia to Australasia
*Lepidozia holorhiza* (Reinw., Blume et Nees) Nees	New for Vietnam	Vietnam	Indochina and Malesia
*Lepidozia miqueliana* Sande Lac.	New for Vietnam	Vietnam	Indochina and Malesia
*Tricholepidozia semperiana* (Steph.) E.D.Cooper	New for Vietnam	Vietnam	South Asia, southern East Asia, then Indochina and Malesia

## Data Availability

The original contributions presented in this study are included in the article. Further inquiries can be directed to the corresponding authors.

## Abbreviations

The following abbreviations are used in this manuscript:

FEB RAS	Far Eastern Branch of the Russian Academy of Sciences
VBGI	Herbarium of the Botanical Garden-Institute FEB RAS, Vladivostok
JNU	Herbarium of the Chonbuk National University, Chonju
